# Unique Structural Fold of LonBA Protease from *Bacillus subtilis,* a Member of a Newly Identified Subfamily of Lon Proteases

**DOI:** 10.3390/ijms231911425

**Published:** 2022-09-28

**Authors:** Alla Gustchina, Mi Li, Anna G. Andrianova, Arsen M. Kudzhaev, George T. Lountos, Bartosz Sekula, Scott Cherry, Joseph E. Tropea, Ivan V. Smirnov, Alexander Wlodawer, Tatyana V. Rotanova

**Affiliations:** 1 Center for Structural Biology, National Cancer Institute, Frederick, MD 21702, USA; 2 Basic Science Program, Frederick National Laboratory for Cancer Research, Frederick, MD 21702, USA; 3 Shemyakin-Ovchinnikov Institute of Bioorganic Chemistry, Russian Academy of Sciences, Moscow 117997, Russia; 4 Institute of Molecular and Industrial Biotechnology, Lodz University of Technology, 90-573 Lodz, Poland

**Keywords:** AAA^+^ proteins, ATPase module, serine-lysine protease, X-ray crystallography

## Abstract

ATP-dependent Lon proteases are key participants in the quality control system that supports the homeostasis of the cellular proteome. Based on their unique structural and biochemical properties, Lon proteases have been assigned in the MEROPS database to three subfamilies (A, B, and C). All Lons are single-chain, multidomain proteins containing an ATPase and protease domains, with different additional elements present in each subfamily. LonA and LonC proteases are soluble cytoplasmic enzymes, whereas LonBs are membrane-bound. Based on an analysis of the available sequences of Lon proteases, we identified a number of enzymes currently assigned to the LonB subfamily that, although presumably membrane-bound, include structural features more similar to their counterparts in the LonA subfamily. This observation was confirmed by the crystal structure of the proteolytic domain of the enzyme previously assigned as *Bacillus subtilis* LonB, combined with the modeled structure of its ATPase domain. Several structural features present in both domains differ from their counterparts in either LonA or LonB subfamilies. We thus postulate that this enzyme is the founding member of a newly identified LonBA subfamily, so far found only in the gene sequences of firmicutes.

## 1. Introduction

ATP-dependent Lon proteases are key components of the protein quality control (PQC) system. PQC plays a leading role in the maintenance of the cellular proteome in organisms belonging to all domains of life, both under normal and stress conditions. PQC system includes families of molecular chaperones and highly selective proteolytic enzymes [1,2,3,4,5]. Chaperones ensure the correct folding and refolding of cellular proteins and the formation of protein ensembles, and also prevent protein aggregation [6,7,8,9]. PQC proteases cleave damaged and abnormal proteins and control the level of regulatory proteins at all stages of the cell cycle [10,11,12,13]. Most chaperones, as well as all proteases of PQC, belong to heat shock proteins (Hsp) [14,15].

PQC proteases are bifunctional, oligomeric enzymes that degrade protein substrates utilizing a processive mechanism (without the release of large intermediates) [16,17,18,19]. Their proteolytic function is coupled to ATP hydrolysis. The protease components are represented by peptide hydrolases of four different classes, while the ATPase components are chaperones of the Hsp100 family belonging to a single superfamily of AAA^+^ proteins (**A**TPases **A**ssociated with various cellular **A**ctivities) [20,21,22,23]. The ATPase and protease components can be either individual subunits, forming heterooligomeric AAA^+^ proteases, or separate domains in a single polypeptide chain of homooligomeric enzymes. All AAA^+^ proteases form barrel-shaped complexes constructed from hexameric rings of AAA^+^ chaperones with a central axial pore, and coaxially docked hexameric or heptameric rings of peptidases forming a degradation chamber with internal active centers. The ATPase component recognizes a protein target to be degraded, remodels it by unfolding, and translocates into the proteolytic chamber for hydrolysis.

Homooligomeric Lon was the first identified ATP-dependent protease, detected during the screening of intracellular peptidases in *Escherichia coli* [24]. Named *Ec*Lon, this enzyme performs more than 50% of selective proteolysis in *E. coli* cells [25]. For many years, *Ec*Lon has served as the principal model used to study general characteristics of ATP-dependent proteolysis.

The *lon* genes from genomes of Gram-positive bacteria *Bacillus subtilis* and *Brevibacillus brevis,* analogous to the *E. coli lon* gene, were isolated and characterized already in the 1990s [26,27]. Sequences of the enzymes encoded by these genes (*Bs*Lon and *Bb*Lon) turned out to be highly similar to the *Ec*Lon: more than 54% of the residues are strictly conserved between *Ec*Lon and *Bs*Lon, or between *Ec*Lon and *Bb*Lon, and the overall similarity between these enzymes is as high as 84%. Primary structures of more than 100 Lon proteases from various organisms became available by the year 2000. Their comparative analysis showed that Lon proteases are non-classical serine peptidases, with catalytic centers formed by serine–lysine dyads [28,29,30].

Two types of peptidase centers (A and B) were noticed in the general pool of Lon proteases, differing in the environment of the catalytically active serine and lysine residues [29,30,31]. The identified differences were accompanied by variations in the architecture of the corresponding ATPase components of Lon proteases, demonstrated earlier [32]. Thus, the Lon family was subdivided into two subfamilies, LonA and LonB.

**Figure 1:**
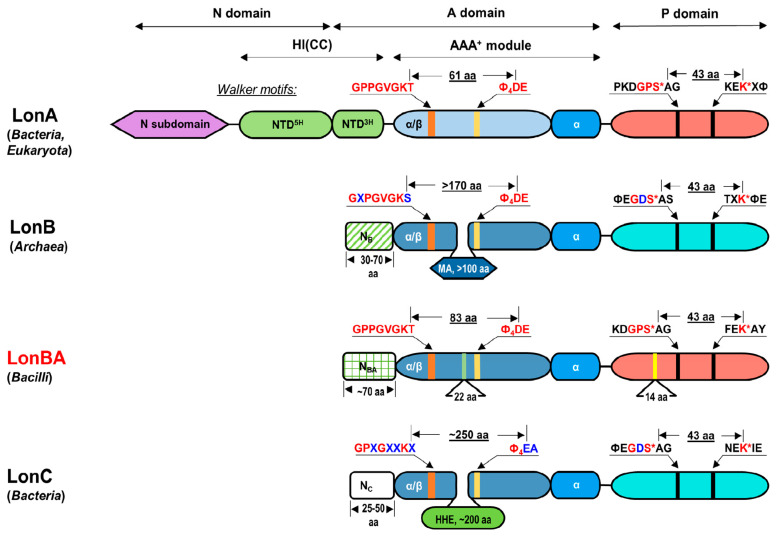
Schematic block diagram of the different subfamilies of Lon proteases. Corresponding functional domains that are more similar to each other are identified by the same color (light blue and blue colors of the α/β subdomains characterize A and B types of AAA^+^ modules, respectively, whereas red and turquoise colors characterize the proteolytic (P) domains of A- and B-type). Consensus fragments of the AAA^+^ modules and P domains crucial to the enzymatic activity are shown together with selected distances between them. Additional sequences in α/β subdomains in LonB and LonC (Membrane Anchoring Subdomain, MA, and Helical Hairpin Extension, HHE) are shown in blue and green, respectively. N_B_, N_BA_ and N_C_ are the N-terminal extensions in proteases of B, BA, and C subfamilies. The N-terminal region of LonAs is formed by three subdomains, with NTD^5H^ and NTD^3H^ forming the so-called helical insertion subdomain, HI(CC) [33].

Definitions of the domain organization of Lon proteases used here follow the recently presented scheme [33] and are shown in Figure 1. A polypeptide chain of all Lon proteases contains an ATPase (A) domain followed by a protease (P) domain, and in some cases also additional domains. The A domain always includes an AAA^+^ module consisting of an α/β and α subdomains, and in some Lons, also additional subdomains (Figure 1).

Members of the LonA subfamily function in the cytoplasm of bacteria and in the mitochondria and chloroplasts of eukaryotes. In addition to the A and P domains, they also include an extended N-terminal region (Figure 1), which contains a fragment of a sequence with a coiled-coil (CC) conformation. The LonB subfamily predominantly consists of membrane-bound Lon proteases of archaea, with the membrane-anchoring (MA) domain embedded in the AAA^+^ module (Figure 1) [30,31]. Crystal structures of the P domains of *Ec*LonA [34] and of *Archaeoglobus fulgidus* LonB (*Af*LonB) [35] revealed their unique fold, highly conserved in both subfamilies [30]. However, although in each subfamily the catalytic Ser and Lys amino acids in the P domains are separated by exactly 42 residues, an important distinction is the presence of a Pro residue before the catalytic Ser in LonA enzymes, whereas the corresponding residue in LonB enzymes is invariably Asp.

Based on unique features of the P domains of Lons observed in the crystal structures, Lon proteases were allocated to the SJ clan in the MEROPS peptide hydrolase database (https://www.ebi.ac.uk/merops/, accessed on 20 May 2022) [36]. Initially, the Lon family was divided into seven identification groups (ID). Bacterial and eukaryotic enzymes of ID groups 001-004 and 006 (75 members) had exclusively A-type active centers, and enzymes from archaea (ID group 005, 14 members) had B-type centers (Figure 1). Some Lon proteases with catalytic centers of both types were combined into a non-characterized ID group 00X (12 members) [30]. The names “LonA peptidase” and “LonB peptidase” were assigned to members of the ID groups 001 and 005, respectively.

Subsequently, another group of enzymes, whose protease and ATPase components resembled their counterparts in LonB proteases, was discovered in thermophilic bacteria [37]. These enzymes also selectively degrade unfolded protein substrates but utilize an ATP-independent mechanism, since they have lost their ATPase activity due to the replacement of some essential residues of ATPase centers. In the MEROPS database, these enzymes are represented as the third Lon subfamily, consisting so far of only eight members, and named LonC (ID S16.007) (Figure 1) [38].

The entire family of Lon proteases currently includes more than 21,000 members, divided into 25 ID groups. However, proteins of many groups either do not contain ATPase components or are non-peptidase homologues of Lon enzymes. True Lons are represented by enzymes of the main groups S16.(001-011) and a few others (for example, groups S16.A(02, 03, 06, 14), with the number of members ranging from 1 to 6). Nevertheless, no new variants of the active centers were identified, with the enzymes having protease centers of either the A or the B type in each ID group.

Recently, the ID group of archaeal LonBs (S16.005) significantly expanded due to the inclusion of some enzymes from bacteria representing Bacilli and Clostridia classes of the phylum Firmicutes [38]. These enzymes bear structural features of both LonA and LonB proteases, and their protease active centers belong to the A type (Figure 1), providing an exception to the current MEROPS classification of Lon proteases. Therefore, they can be assumed to represent a separate “hybrid” LonBA subfamily in the Lon family. We describe here the isolation, initial characterization, and a crystallographic and modeling study of the first representative of the proposed new subfamily—LonBA protease from *B. subtilis*.

## 2. Results and Discussion

### 2.1. Analysis of the Primary Structures of Lon Proteases Belonging to ID Group S16.005

The ID group S16.005 (LonB peptidases) in the MEROPS database currently includes 145 members. Out of these, only 19 enzymes (13.1%) are LonB proteases of archaea, 40 (27.6%) are represented by enzymes from Bacilli, and 86 (59.3%) are from Clostridia. True archaeal proteases of this group are formed by ATPase and protease domains of B-type, and their AAA^+^ modules include an inserted MA domain with a transmembrane fragment (Figure 1) [30,39]. The amino acid sequences of bacterial enzymes of this ID group lack both the extended N-terminal region characteristic of LonAs, as well as the large, inserted MA domain inherent in LonBs. Nevertheless, their ATPase domains demonstrate a number of similarities with the ATPase domains found in both subfamilies of Lons, but their protease domains are more similar to A-type than to B-type Lons, which implies a “hybrid” domain organization of this new group of enzymes (Figure 1).

Despite the fact that the presence of a Lon protease in *B. subtilis*, which contains neither an N-terminal region as in LonA, nor a membrane anchor as in LonB, has been previously noted [39,40], this observation has not been followed up. There is no information in the literature on the isolation and study of Lon proteases from Clostridia that are assigned to ID groups S16.001 and S16.005. For these reasons, we selected *B. subtilis* as a source for purification and study of a model bacterial “hybrid” Lon protease (*Bs*LonBA) of the S16.005 group.

The primary structures of LonA and LonBA proteases from eight bacillary sources were compared with the structure of the model enzyme, *Ec*LonA (Figure S1). LonBAs were found to contain two functional domains (AAA^+^ module and P domain) connected by a 13- or 15-residue-long linker, as well as an N-terminal fragment consisting of 72 or 73 residues. Similarity levels within each fragment of the LonBA sequences were evaluated. It was found that the most conserved is the α/β subdomain of the AAA^+^ module (Figure 1), with a total sequence similarity of 90.0% (with 69.4% of strictly conserved residues), whereas the least conserved was the α subdomain (53.3% similarity and 27.8% identity). The N-terminal extension and the P domain show similar conservation: 75.3% similarity and 26.4% identity (for the N fragment), 71.1% similarity, and 38.5% identity (for the P domain).

According to the prediction (http://distillf.ucd.ie/porterpaleale/, accessed on 20 May 2022), the N-extension of the *Bs*LonBA is predominantly α-helical (Figure S1). On that basis, we compared this fragment with helical subdomains NTD^5H^ and NTD^3H^ of LonA proteases. It was found that the LonBA N-fragments do not resemble either the NTD^3H^ subdomain (Figure S1) or the NTD^5H^ subdomain (not shown). There is also no sequence similarity between the N-terminal fragments of LonBAs and the corresponding fragments of LonB and LonC proteases (Figure S2).

While comparing the AAA^+^ modules of LonA and LonBA proteases, a distinction in size of the N-terminal α-helix, as well as differences in the distances between the enzyme consensus fragments, may be detected (Figure S1). In particular, in LonA proteases, the conserved Asp residue of the Walker B motif is separated from the Lys of the Walker A by 60 residues, whereas in LonBA proteases, this distance increases to 82 residues due to insertions and deletions that are localized before, after, and/or within Pore loop-1 (Figure S1). Other insertions or deletions are found in the loops connecting conserved secondary structure elements, and they are discussed in more structural terms below. On the contrary, the α subdomains of LonBA proteases are smaller than in LonAs, due to deletions of some residues in the regions of the first and third α helices (Figure S1).

Although the overall similarity between the P domains of LonAs and LonBAs is high and the distances between their catalytic residues (Ser 445 and Lys488 in *Bs*LonBA; Ser679 and Lys722 in *Ec*LonA, see Figure S1) are identical, the presence of an inserted fragment of 14 amino acids, which is embedded in a region located before the first α helices of the P domains of LonAs (E632-A647 in *Ec*LonA), is significant (Figure S1). It can be noted that the P domains of bacillary LonAs and LonBAs do not contain any cysteine residues and therefore cannot form an internal disulfide bond, which is characteristic for *Ec*LonA [34]. However, as will be shown below, the presence of the disulfide in the latter enzyme does not affect the conformation of its region compared to its counterpart in *Bs*LonBA.

Thus, a comparison of the amino acid sequence of LonBA with the sequences of LonA, LonB, and LonC proteases suggests noticeable differences between these Lon subfamilies. We used the *Bs*LonBA enzyme as a model representative of that proposed novel subfamily. The enzyme was cloned, expressed, and its basic enzymatic properties were studied. Additionally, the P domain of *Bs*LonBA was crystallized and its structure was compared to structures of P domains of other members of the Lon family.

### 2.2. Preparation, Limited Proteolysis, and Preliminary Characterization of the Recombinant BsLonBA

Full-length *Bs*LonBA was cloned and expressed in *E. coli* as a C-terminal His_6_-tagged protein (*Bs*LonBA-H6), which appeared to be soluble only in the presence of detergents (CHAPS or DDM). To identify the domain organization of the enzyme, we used limited proteolysis by chymotrypsin, which previously allowed us to successfully determine the domain boundaries in *Ec*LonA and study its nucleotide-dependent conformational changes [41]. Three main cleavage sites that led to the formation of stable fragments were identified (Figure S1). However, these sites (F238/R239, Y298/T299, and W331/V332) turned out to be of limited usefulness for fixing domain boundaries, since they are located in the α/β subdomain before its consensus sensor-1 (s1) residue, as well as in the central part and in the C-terminal fragment of the α subdomain, respectively. In addition, unlike *Ec*LonA, limited proteolysis of *Bs*LonBA was not affected by either ATP or Mg^2+^ ions. Cleavage of *Bs*LonBA within the AAA^+^ module may indicate its partial disorder, presumably due to the insertions and deletions noted above. On the other hand, the absence of limited proteolysis sites within the region corresponding to the *Ec*LonA P domain (residues 353–552 in *Bs*LonBA) demonstrates its structural stability.

In terms of enzymatic properties, the newly isolated *Bs*LonBA-H6 protease is radically different from the “classical” *Ec*LonA. The latter enzyme, which also bore a C-terminal hexahistidine tag, was shown to hydrolyze ATP effectively, and, in the presence of a protein substrate, the ATPase activity increased significantly [42,43]. However, *Bs*LonBA-H6 does not show the ability to hydrolyze ATP either in the absence or in the presence of a substrate protein.

The proteolytic activity of both Lon proteases was monitored through hydrolysis of β-casein. In the presence of the ATP-Mg^2+^ *Ec*LonA-H6 degrades a protein substrate utilizing the processive mechanism, but in the absence of a nucleotide, the enzyme exhibits weak non-processive activity (Figure S3A). In both cases, bortezomib, an organoboronic serine protease inhibitor, completely suppresses the proteolytic function of *Ec*LonA-H6. The *Bs*LonBA-H6 protease does not cleave β-casein in the time interval in which the proteolytic activity of *Ec*LonA-H6 was tested. However, when the reaction time is increased to 24 h or more, products of non-processive hydrolysis of the protein substrate are detected, both in the presence of ATP-Mg^2+^ and in the absence of effectors (Figure S3B). In addition, hydrolysis of the target protein is accompanied by noticeable autolytic degradation of the enzyme (Figure S3B). Thus, under the conditions utilized in this study, *Bs*LonBA-H6 exhibits very low non-processive proteolytic activity, and at the same time bortezomib practically does not affect the function of the enzyme (Figure S3B).

The results presented above show that *Bs*LonBA-H6 is significantly inferior to *Ec*LonA-H6-protease in both the ATPase and proteolytic activities. However, peptidase activities of these enzymes are quite comparable. It was found that basic peptidase activities (the hydrolysis efficiency of PepTBE in the absence of a nucleotide) of *Bs*LonBA-H6 and *Ec*LonA-H6 differ by no more than 5–6 times (Figure S4, lines 1 and 3). Bortezomib exhibited an inhibitory effect on the peptidase activity of both enzymes (Figure S4, lines 2 and 4), but with different efficacy: the IC_50_ values differ by more than 20 times (0.05 μM for *Ec*LonA-H6 and 1.15 μM for *Bs*LonBA-H6). The presence of the ATP-Mg^2+^ complex leads to the activation of the peptidase activity of *Ec*LonA-H6, but does not affect peptide hydrolysis by *Bs*LonBA-H6, which is consistent with the absence of its ATPase activity.

Differences in the ATPase and proteolytic activities of *Bs*LonBA and *Ec*LonA may indicate that the formation of a functionally active conformation of the AAA^+^ module of the former may require either optimization of the enzyme isolation method or a search for a putative adaptor protein, not currently identified. Important effects of adapter proteins were previously shown, for example, for ClpCP [44,45]. A search for conditions that promote the formation of the functionally correct active structure of *Bs*LonBA-H6-protease remains a future direction in the study of enzymes of this new Lon subfamily.

### 2.3. Crystal Structure of the P Domain of BsLonBA

**Figure 2:**
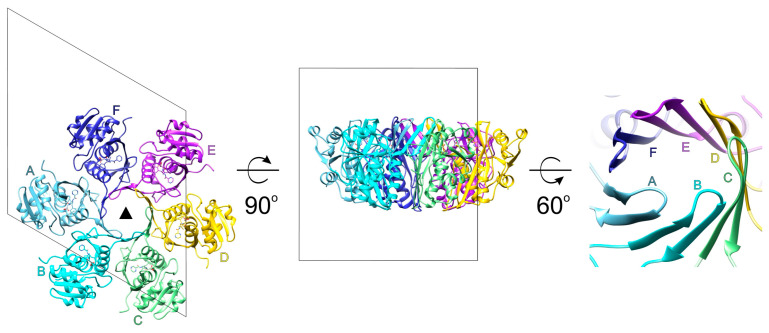
Crystal packing of the protease domain of *Bs*LonBA. Unit cell of *Bs*LonBA crystals with six chains of the protease domain of *Bs*LonBA organized around the crystallographic threefold symmetry axis depicted as a black triangle. The right panel shows the close-up view of the 12-stranded β-barrel created by hairpins β4–β5 of the six protease domains labeled A–F.

The crystal structure of the P domain of *Bs*LonBA (residues 347–552) was determined at the resolution of 1.9 Å. Crystals belong to the hexagonal space group *P*6_3_ with two molecules (designated A and B) in the asymmetric unit, arranged around the crystallographic 3-fold axis. This arrangement results in the creation of an expected planar hexamer (Figure 2). Such a hexamer is similar to the hexameric P domains of *Ec*LonA and *Af*LonB, to the P domains in the almost full-length hexameric proteases LonB from *Thermococcus onnurineus* (*Ton*LonB) and LonC from *Meiothermus taiwanensis* (*Mta*LonC), as well as in cryoEM structures of full length human mitochondrial LonA (*h*MtLonA) (as recently reviewed [33]). It must be stressed, however, that the protein used for crystallization behaved as a dimer when run on a sizing column before crystallization experiments were set up. The fact that an isolated P domain of *Bs*LonBA emulates other Lons, recreating hexamers in the crystal lattice, leads to an assumption that the structure of *Bs*LonBA P domain mimics functional, hexameric organization of this protein. Similarly to other Lons, in the *Bs*LonBA oligomer six P domains have a planar arrangement with the proteolytic chamber in the center. However, one structural element strikingly differentiates *Bs*LonBA from other known Lons. The insertion of 14 residues (392–405) in the P domain of *Bs*LonBA (Figure S1) creates a 12-stranded β-barrel in the core of the hexameric assembly formed by β-hairpin inserts of the neighboring subunits (Figure 2; a detailed description below).

**Figure 3:**
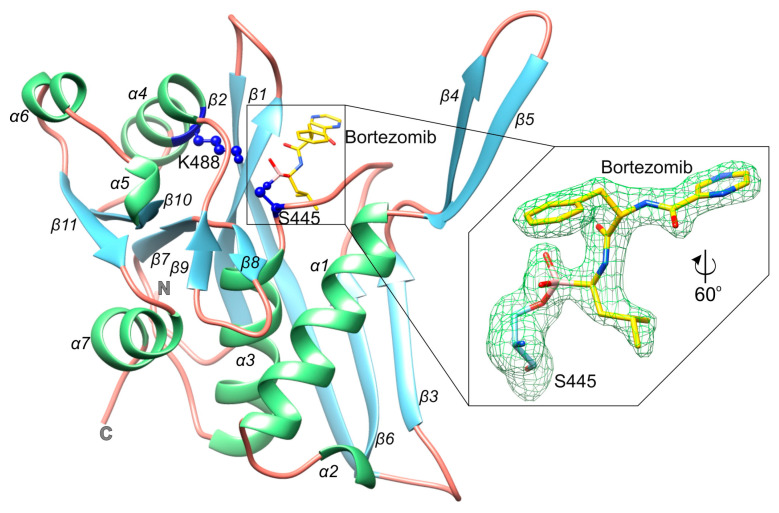
Structural details of the protease domain of *Bs*LonBA. Cartoon representation of the protease domain of *Bs*LonBA inhibited by bortezomib. Structure is color-coded by the secondary structure elements: helices (green), β-strands (light blue), coil regions (salmon); the catalytic dyad residues (S445 and K488) are marked with blue; right panel shows bortezomib bound to the catalytic S445 with displayed *F_o_*-*F_c_* omit map contoured at 3*σ* level (green mesh).

The overall fold of the P domain of *Bs*LonBA resembles the equivalent domains of enzymes belonging to A, B, and C subfamilies of Lon proteases. In *Bs*LonBA it consists of a single polypeptide chain of ~200 amino acids, which forms 11 β-strands and seven helices (Figure 3 and Figure S2). The molecule can be divided into two subdomains with a linking fragment comprising residues 460–467. The core of the first subdomain is built of a 4-stranded β-sheet and three helices. A mixed β-sheet consisting of an N-terminal β-hairpin (β1–β2), β6, and β3 forms the back wall. The protruding part of the subdomain is formed by another β-hairpin (β4–β5), which participates in the formation of the central β-barrel of the hexamer. It precedes in the sequence the longest helix α1, which runs on the front side of the mixed β-sheet. Helix α1 and a short helical turn α2 (directly preceding β6 in the sequence) participate in the inter-protomer interactions with the back wall of the neighboring unit of the hexamer. Helix α1 also interacts with helix α3, which runs in the central part of the P domain and leads to the second subdomain. This subdomain includes a central three-stranded parallel β-sheet (β7–β10–β11) flanked by helix α7 from the bottom side and by helices α4 and α6 from the top side. A short helix α5 directly follows strand β10. The subdomain has also an extended loop with two very short β-strands (β8–β9), which are placed above the N-terminal side of the α3 and protrude towards α1, participating in interactions with the neighboring protomer.

**Figure 4:**
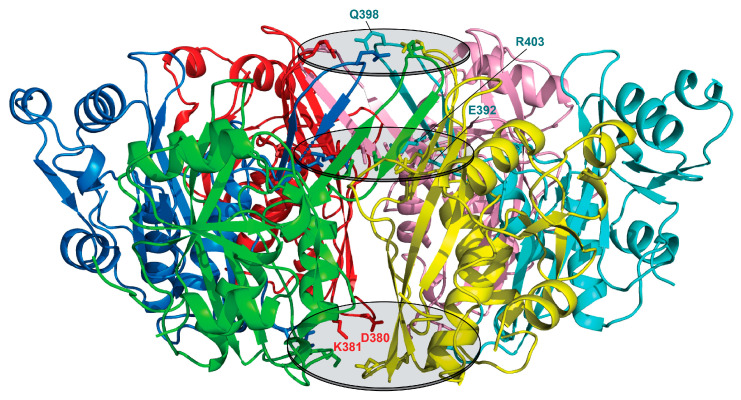
Hexamer of the protease (P) domain of *Bs*LonBA (side view). Six protomers of the protease domain forming a hexamer of *Bs*LonBA are shown in different colors. Three layers of charged or hydrophilic residues from each protomer form a planar arrangement (marked with shaded ovals) with their side chains (shown in sticks) oriented into the central chamber of a hexamer.

**Figure 5:**
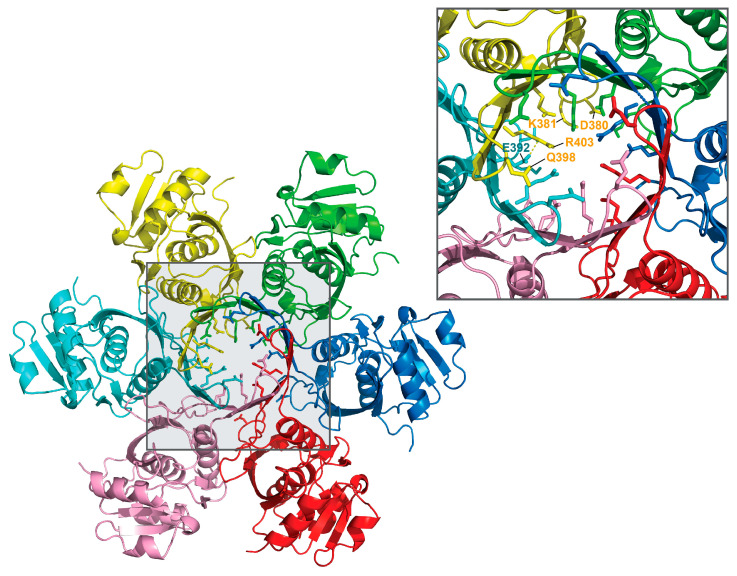
Hexamer of the protease domain of *Bs*LonBA (top view). Top view of the central chamber of the P domain with protruding side chains of the residues in an extended conformation (shown in sticks).

The unique β4–β5 inserted hairpin in *Bs*LonBA (392–405) is created by two five-residue-long strands composed mainly of polar residues and a four-residue-long connecting loop. Six inserts arranged around the three-fold symmetry axis complete a 12-stranded antiparallel β-barrel, where each insert forms nine hydrogen bonds with the neighboring subunit. In the central core of this barrel, two layers of charged or hydrophilic residues from each protomer form a planar arrangement with their side chains in an extended conformation and oriented towards the center of the barrel (Figure 4, top and middle ovals shaded gray). The top layer is formed by the side chains of Gln398. The width of the barrel opening within this layer is ~11 Å (Figure 4 and Figure 5). The second layer is formed by the side chains of conserved Glu392 and Arg403, that are poised towards the interior of the barrel, creating a second lid with a ~8 Å wide opening. An ion pair between the Nε atom of Arg403 side chain and Oε1 atom of Glu392 from two neighboring protomers seems to support the extended conformations of their side chains and their orientations towards the center of the barrel (Figure 5). The third layer is formed at the bottom of the central chamber of the P domain hexamer by the side chains of Asp380 and Lys381 from the same protomer, which are also oriented towards the center with the distance between their charged groups ~3.4 Å (Figure 4 and Figure 5). Here, the chamber widens to ~16.5 Å.

### 2.4. The Mode of Binding of Bortezomib to the P Domain of LonBA and Other Lon Proteases

**Figure 6:**
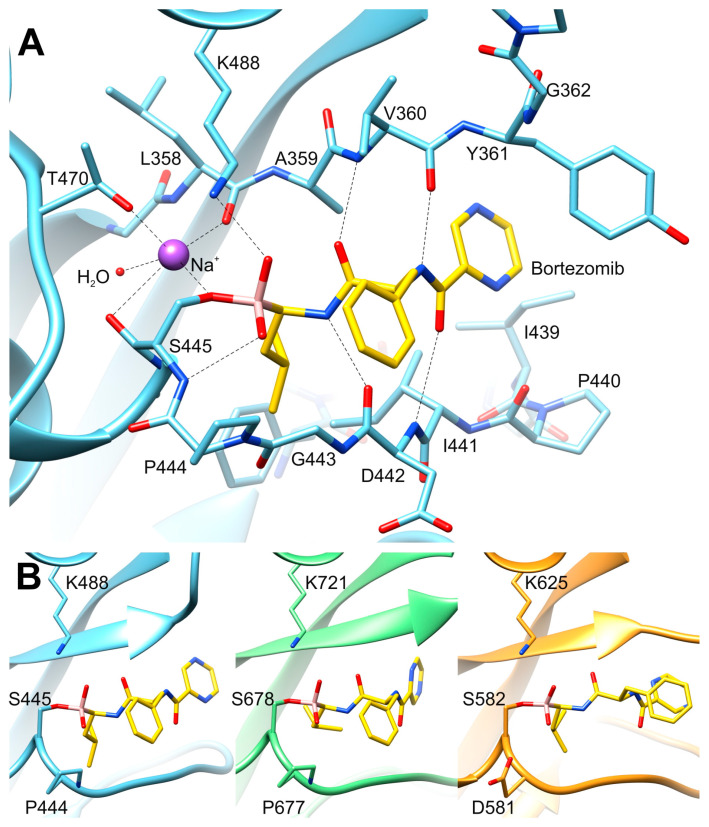
Proteolytic active site of Lon proteases inhibited by bortezomib. (**A**) Structural details and major interactions of bortezomib and sodium ion bound to the active site of *Bs*LonBA; (**B**) Side-by-side comparison of bortezomib inhibiting the active sites of various Lon proteases: the structures of *Bs*LonBA, *Mta*LonA (4ypm), and *h*MtLonA (6x27) are shown from left to right.

We have captured the P domain in an inhibited state, in the complex with bortezomib, a covalently bound inhibitor. Unambiguous electron density maps (Figure 3) allowed us to determine the conformation of the inhibitor within the substrate-binding site. This site is created by a β-hairpin (β1–β2), a long loop connecting β6 and α3, and the protruding loop made of β8–β9. The catalytic Ser445 is located in the sequence just before α3, whereas Lys488, the other member of the catalytic dyad, is located within helix α4 (Figure 3). Such an architecture that includes several flexible structural elements allows accommodatinf a variety of conformations. Bortezomib is covalently bound to the Oγ of Ser445 via the boronate moiety. The inhibitor structurally mimics a peptide substrate with leucine and phenylalanine side chains, as well as pyrazinoic acid moiety. Within the active site, bortezomib creates β-sheet-like interactions with residues of β1 (A359, V360, Y361) on one side, and residues of the loop β6-α3 (I441, D442) on the other side (Figure 6A). Oxygen atoms of the boronate moiety make additional hydrogen bonds with the side-chain amine of Lys488 and the main chain nitrogen of Ser445. Analysis of the vicinity of the active site identified a putative sodium cation coordinated by carbonyl oxygens of Ser445 and Leu358, Oγ atoms of Thr470 and Ser445, and a water molecule (Figure 6A). The location and conformation of the inhibitor are similar to its counterparts in other Lon proteases (see below) (Figure 6B).

High-resolution crystal structures that include bortezomib-inhibited protease domains are available for *h*MtLonA (PDB ID 6x27), LonA from *M. taiwanensis* (*Mta*LonA) (4ypm), and *Mta*LonC (4fwd) [33]. There are currently no published data available for any LonB complexed with any ligand, including bortezomib. Among the available structures of different protease domains of LonB, *Af*LonB (exemplified by 1z0w) has an active site area that is significantly different from what is expected for an enzyme with no ligand bound, and LonB from *Methanocaldococcus jannaschii* (*Mj*LonB, 1xhk) is in a self-inhibited conformation, in which Asp547, separated by two residues from the catalytic Ser550, occupies a part of the substrate-binding site. Although *Ton*LonB (3k1j) has both the active site serine and lysine replaced by alanines, its substrate-binding site is not occupied. This enables the active site to adopt an open conformation for accepting a ligand, allowing direct comparison with *Bs*LonBA inhibited by bortezomib.

The P domain of *h*MtLonA (6x27) can be superimposed on its *Bs*LonBA counterpart with an rmsd of 1.34 Å for 155 Cα atoms, whereas the superposition of *Mta*LonA (4ypm) yields an rmsd of 1.54 Å for 146 Cα atoms, despite the amino acid identity compared to *Bs*LonBA being larger (41%) for the latter enzyme than 35% for the former. The overall similarity of the P domains of *Bs*LonBA and *Ton*LonB (3k1j) is very high (1.34 Å for 145 Cα atoms, 43.5% identity). Although the identity of 24.3% is lower compared to *Mta*LonC (4fwd), the fold of these proteins is still very similar, with an rmsd of 1.53 Å for 156 Cα atoms.

The boronate group of bortezomib is covalently bound to the active site serine and the conformation of its main chain is virtually identical in all compared structures (Figure 6B). The extended inhibitor mimics a β strand and makes a number of hydrogen bonds with the main chain peptides of the two adjacent chains. The side moieties of bortezomib occupy the S1–S3 subsites of the substrate-binding crevice. In the structure of the *h*MtLonA complex (6x27), the P2 phenyl group of the inhibitor has two conformations, whereas its conformation in *Mta*LonC (4fwd) corresponds to the alternate conformation in 6x27 (not shown). This subsite is located on the surface of the enzyme and it is rather open to solvent.

Each of the four structures of Lon proteases complexed with bortezomib (listed above) contains two fully conserved solvent molecules in the immediate vicinity of the active site serine side chain. The distance between the Oγ atom of the catalytic serine and the first one of these solvent molecules varies in these structures between 2.62 and 2.88 Å, but this solvent molecule was annotated as water in *h*MtLonA (6x27) and *Mta*LonC (4fwd), as Mg^2+^ ion in *Mta*LonA (4ypm), and now as Na^+^ in *Bs*LonBA. This molecule is within a hydrogen bond distance to three main chain carbonyl atoms, as well as to another conserved solvent molecule, invariably identified as water. The presence of five potential ligands of these solvent molecules, as well as the geometry of their interactions, makes it unlikely that they could be waters, since a water molecule could not be a hydrogen bond donor to three oxygens acting as acceptors. The distances between this molecule and its neighbors are significantly longer than the coordination distances expected for a Mg^2+^ ion, and the geometry of interactions is also not octahedral. Only the postulated Na^+^ ion could be reconciled with the observed geometry, since that ion is quite frequently found to be coordinated to five oxygen atoms [46]. This interpretation makes the previous proposal that a magnesium ion might be directly responsible for the transition between the inactive and active conformation of the active site of Lon rather unlikely.

A defining feature of the P domains of LonA proteases is the identity of the residues preceding the catalytic serine (Ser679 in *Ec*LonA), which is always a proline, whereas the corresponding residue is an aspartic acid in LonBs and LonCs. Nevertheless, the main-chain torsion angles are virtually identical in the presence of bound inhibitors regardless of the nature of this residue (Figure 6B), making it unlikely that its identity plays a major role in establishing the catalytic properties of the protease domains of different subfamilies of Lon proteases.

### 2.5. Comparison of the BsLonBA P Domain with Its Counterparts in Other Lon Proteases

**Figure 7:**
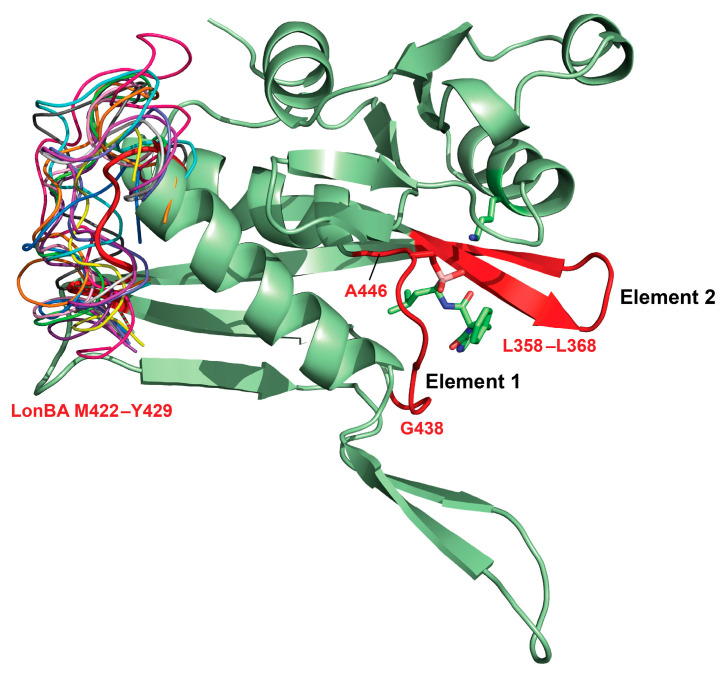
Mobile areas of the P domains of Lon proteases. Overall cartoon structure of the *Bs*LonBA P domain (pale green) is used to highlight the mobile areas in various Lons. The first mobile area is a flexible surface loop (residues 422–429 in *Bs*LonBA) with different lengths and conformations in all subfamilies of Lons from different sources (color coded). The other two mobile areas involve a fragment containing the catalytic Ser residue (residues 438–446 in *Bs*LonBA, Element 1 shown in red) and an adjacent β-loop comprising residues 358–368 (Element 2 shown in red). The inhibitor, bortezomib, is shown in sticks.

**Figure 8:**
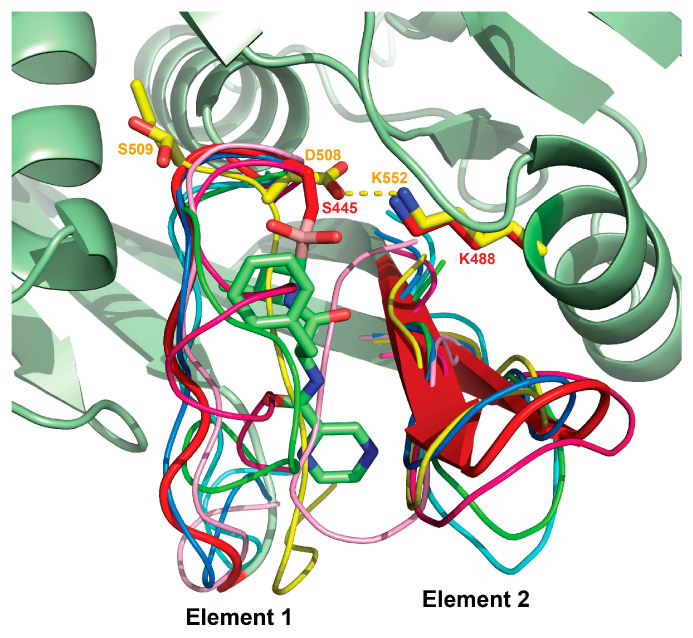
Transition from the active to the inactive state of the P domain. This transition is assisted by the movement and conformational changes in two mobile elements (1 and 2) in the active site. These superimposed fragments are shown in *Bs*LonBA (red); LonAs: *Ec*LonA (1rre and 6u5z, pink and green, respectively) and *h*MtLonA (7krz, cyan); LonBs: *Mj*LonB (1xhk, magenta), *Ton*LonB (3kij, blue), and *Af*LonB (1z0w, yellow). The inhibitor, bortezomib, bound to *Bs*LonBA is shown in sticks, the P domain of *Bs*LonBA is shown in a cartoon representation in pale green.

In addition to the structure of the P domain of *Bs*LonBA described here, six structures of its LonA counterparts, three of LonB, and one of LonC are currently available [33]. All of them start with long β-hairpins comprising strands β1 and β2 (Figure 3). Strand β1 participates in the formation of the ligand-binding site with residues interacting directly or via solvent with the bound inhibitor (Figure 6). The conformation and position of the tip of the β1–β2 loop vary in different Lons according to the functional state of the individual P domains, as well as the presence or absence of a bound ligand (Figure 7 and Figure 8).

**Figure 9:**
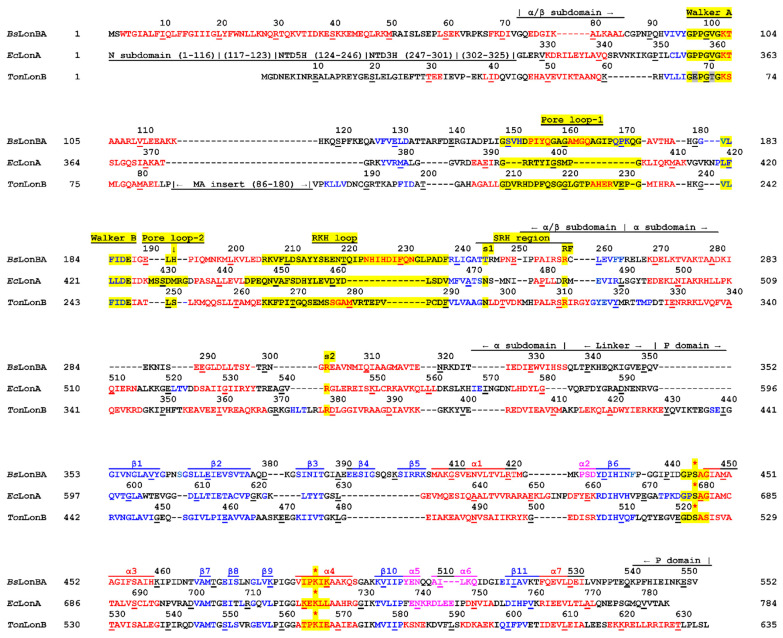
Structure-based sequence alignment of *Bs*LonBA, *Ec*LonA, and *Ton*LonB. Domain organization of Lon enzymes is shown. Red indicates amino acids that form α helices, magenta–3_10_ helices, blue–β strands, and black color indicates amino acids that are not included in the regular secondary structure elements. Consensus sequence elements are highlighted in yellow: Walker motifs A and B, Pore loop-1, Pore loop-2, RKH loop, SRH region, the residues sensor-1 (s1), sensor-2 (s2), and arginine finger (RF), as well as fragments containing the catalytic serine (S*) and lysine (K*) residues.

The loop connecting strands β2 and β3 is one-residue longer in *Bs*LonBA than in *Ec*LonA, and two-residue shorter than in *Ton*LonB (Figure 9). *h*MtLonA (6x27) has the longest insert in this loop compared to bacterial LonAs, as well as LonBs (~10 residues), which forms a flexible loop disordered in the structure. In *Bs*LonBA, residues Asp380 and Lys381 of this loop in each protomer form the planar arrangement of their side chains in an extended conformation at the bottom of the central chamber of the P domain hexamer, as described above (Figure 4 and Figure 5). Interestingly, *Ec*LonA and *Mj*LonB (1xhk) also have topologically equivalent corresponding residues within this loop (Lys621 in *Ec*LonA and Lys487 in *Mj*LonB).

Four β strands (β1, β2, and β3, as well as β6, positioned between β2 and β3 (Figure 3)), form in *Bs*LonBA a β-sheet that is also conserved in all Lon subfamilies. Unique among all subfamilies of Lon, only *Mta*LonC includes a helical twist after β3. In turn, *Bs*LonBA is the sole exception that includes a protruding β-hairpin (β4–β5) that forms a β-barrel in the central part of the proteolytic chamber of the hexamer (Figure 2 and Figure 3). No other Lon with known structure has a similar insert that would correspond to β4–β5 of *Bs*LonBA (Figure S2). The orientation of the longest helix α1 is slightly different in other known Lons than it is in *Bs*LonBA. Additionally, the loop that follows, connecting helix α1 with strand β6, is the most variable region in different Lons (Figure 7). This loop can be longer, as in *Mj*LonB, with no helical twist, or it may form two small helices, as in *Mta*LonA or *Bs*LonBA. However, a common feature of this loop is that it creates a helical twist just before β6 (*Bs*LonBA, LonA protease from *Yersinia pestis* (*Yp*LonA), *Mta*LonA, *h*MtLonA, *Ton*LonB, *Af*LonB) (shown for *Bs*LonBA in Figure 3). The long flexible loop connecting strand β6 with helix α3 in *Bs*LonBA carries the catalytic Ser445 (Figure 3). After that, the protruding region consisting of β7, as well as of the loop that comprises strands β8 and β9 and leads to helix α4 is structurally very similar in all Lons, with helix α4 carrying the catalytic Lys488 (Figure 3). The only exception is again *Mta*LonC, where α4 is longer and ends with an additional inserted loop created by seven residues (Figure S2). The region that follows (β10-α5) is rather similar in various Lons, although *Bs*LonBA is characterized by a break in a shorter helix α5, which is followed by α6 and then β11. LonAs and LonC are characterized by a longer helix α5 and an insertion that follows it. This insertion forms a helix and a loop that leads to β11. In LonBs, there is no additional helix after α5. *Af*LonB has a long loop, whereas this loop is significantly shorter in *Mj*LonB. After β11, the common feature of all Lons is the presence of α7 and some of them end with another helix close to the C terminus (*Ton*LonB, *Af*LonB, and *Mta*LonC).

Comparison of the structure of P domain of *Bs*LonBA with the structures of the corresponding domains in LonAs and LonBs, determined in either single-domain or multi-domain constructs, reveals a similar mechanism of transition of the state of the active site from an active conformation to an inactive one. Two elements of the active site area embracing the ligand binding area, which include on one side a flexible loop (residues 438–446 in *Bs*LonBA, element 1) and on the other side a β1–β2 loop (residues 358–368, element 2, Figure 7), would change their conformation and mutual arrangement, yielding a particular functional state of the P domain (Figure 8). Thus, in the structure of *Bs*LonBA, the active site of the P domain adopts an open conformation, with bortezomib bound in the center and flanked by both mobile elements listed above. In both the crystal structure of the P domain of *Ec*LonA and cryo-EM structure of the full-length enzyme, their uninhibited active sites are found in an inactive conformation, although the mechanism to achieve this state was different. In the crystal structure of an isolated P domain (1rre), the fragment comprising element 2 moved to occupy the binding site area (Figure 8, shown in pink), whereas in the cryo-EM structure of the full-length enzyme (6u5z) a fragment comprising element 1 moved to occupy the same space, leaving the position of element 2 unchanged (Figure 8, shown in green). In the structure of *h*MtLonA (7krz, Figure 8, shown in cyan), the inhibitor, bortezomib, is bound in the active site, and the flanking elements remain in an open conformation, leaving the active site area undisturbed.

Although in the crystal structure of an unliganded, isolated P domain of *Mj*LonB (1xhk, Figure 8, shown in magenta), the inactive state is supported by the shift of element 1 to occupy the active site area, in the crystal structure of the almost full-length *Ton*LonB (3kij, Figure 8, shown in blue), the active site area assumes an open conformation even in the absence of a ligand.

While the mechanism of transition from the active to inactive state of the P domain that is assisted by the movement and conformational changes in two mobile elements of the active site seems to be consistent for several LonAs and LonBs, one example completely stands out. Significant differences were observed in the active site of an isolated P domain of *Af*LonB (1z0w, Figure 8, shown in yellow), where the part of the chain around the catalytic Ser509 was flipped away from the helix carrying the other catalytic residue, Lys552. As a result, the catalytic dyad no longer exists, and a fragment comprising element 1 moves to occupy the substrate binding site, establishing an inactive state of the enzyme. We can speculate that such a conformational change could be facilitated by the substitution of the conserved proline residue before the catalytic Ser in LonAs by the aspartate in LonBs, making such a dramatic flip of the main chain around this residue possible.

### 2.6. Model Building of the ATPase Domain of BsLonBA

**Figure 10:**
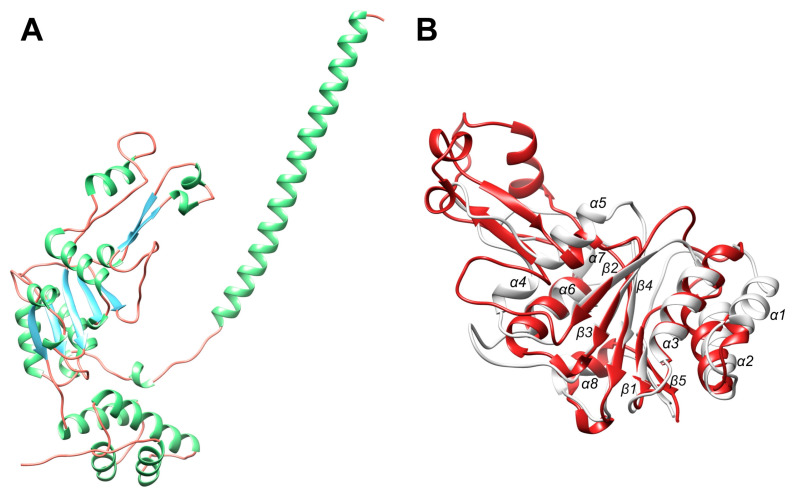
Topology of the A domain of *Bs*LonBA. (**A**) Model of the A domain of *Bs*LonBA based on structure prediction with AlphaFold2. Colors: α-Helices green, β-strands blue, irregular structure red. (**B**) Superposition of the core of the model of the A domain of *Bs*LonBA shown in panel A (red) and the AAA^+^ module of the *Ec*LonA (6u5z, white).

Although we have not determined the structure of the AAA^+^ module of *Bs*LonBA experimentally, a putative model was obtained with the prediction program AlphaFold2 [47] (Figure 10A). It was our expectation that some features that specifically differentiate LonBAs from other members of this family might be important in deciding whether *Bs*LonBA should be considered as a representative of a new subfamily of Lons. The original division of the Lon family into the subfamilies LonA and LonB was based, at least in part, on the differences in the location of the enzymes in the cells. Whereas LonA is a soluble cytoplasmic or mitochondrial enzyme, LonB was found to be membrane-associated [30,48,49]. The fragment of LonB that carries the membrane-insertion sequence is located in the α/β subdomain between the Walker motifs A and B and consists of over 100 amino acids, some of which assume the helical conformation necessary for insertion into cell membranes. There is no corresponding insertion in the sequence of *Bs*LonBA, although that enzyme may also be described as being membrane-associated according to programs that search for transmembrane fragments (including SMART, TMHMM, HMMTOP, TMDAS and others; see Materials and Methods). In this case, however, the membrane-insertion fragment is most likely located at the N terminus of the sequence. The model calculated by AlphaFold2 included a very long α helix (consisting of 52 amino acids) at the N terminus (Figure 10A), with the first ~20 residues being hydrophobic and thus capable of being inserted into a membrane.

### 2.7. Comparison of the Functionally Important Elements in the AAA^+^ Module of BsLonBA with Their Counterparts in LonA and LonB

An overall comparison between the structures of the AAA^+^ modules of *Bs*LonBA and the corresponding fragment of *Ec*LonA (6n2i) yields an rmsd of 2.7 Å for 195 Cα pairs, with sequence identity 18.5% and 19 gaps. An analogous comparison with the AAA^+^ module of *Ton*LonB (4zpx) yields an rmsd of 2.7 Å for 214 Cα pairs, with sequence identity 23.8% and 15 gaps.

The AAA^+^ module of Lon proteases belongs to the HCLR clade, the name of which is derived from the four families that also include HslU/ClpX, ClpABC-CTD, and RuvB [50]. It differs from the simplest module, such as that found in the clamp loader clade [48] (exemplified by the structure with PDB ID 1njg [51]), by the presence of a β loop insertion that emerges from a single helix in the clamp loader, breaking the corresponding helix in Lon into two small helices: α4 and α5 (Figure 10B). From here on, a new numbering of the secondary structure elements for the A domain is used (Figure 10B). A set of conserved consensus fragments of the AAA^+^ module includes Walker motifs A (residues 356–363) and B (419–424), as well as residues forming sensor-1 (Asn473), sensor-2 (Arg542), and the arginine finger (Arg484), in *Ec*LonA numbering. The corresponding residue numbers for *Ton*LonB and *Bs*LonBA are presented in Figure 9.

**Figure 11:**
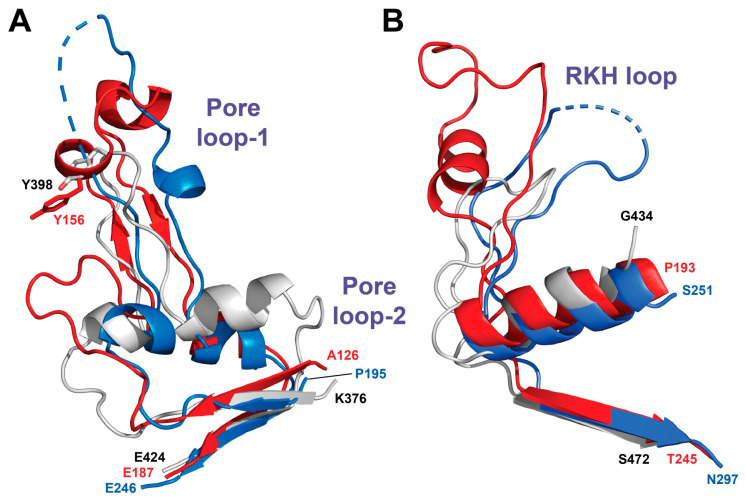
Superposition of three different Pore loops in various Lon subfamilies. Pore loop-1 (**A**) and RKH loop (**B**) are longer in both *Bs*LonBA (red) and *Ton*LonB (blue) than the corresponding loops in *Ec*LonA (white), while Pore loop-2 (**A**) is longer in the latter. Dashed lines indicate fragments that are not modeled in the experimental structures due to the lack of electron density.

The central pore of a hexamer of Lon is lined with axial loops, required for protein unfolding and translocation. Three different pore loops, called Pore loop-1, Pore loop-2 and RKH, are found in almost all AAA^+^ unfoldases. They interact with polypeptide substrates and pull them into the pore in a concerted way, with nucleotide-dependent loop movements unfolding those substrates that otherwise cannot enter the pore. Pore loop-1 is involved in polypeptide translocation and consists of 11 residues in *Ec*LonA (6n2i; [52]), 24 residues in *Ton*LonB (4zpx; [53]), and 25 residues in the predicted structure of *Bs*LonBA (Figure 9). According to the model this insert in *Bs*LonBA resembles more closely the one that is present in LonB than in LonA (Figure 11A). Although the estimate of the size of this insertion is fairly accurate, the predicted helical turns at the tip of the loop may not be necessarily modeled properly in *Bs*LonBA due to the lack of a proper template, since that part of the loop is disordered in *Ton*LonB.

While in enzymes belonging to all Lon subfamilies the insertion of Pore loop-1 breaks a single helical fragment into two small helices (α4 and α5; but in the model of LonBA α4 is only a helical turn, Figure 10B), the axis of the second small helix that follows the insertion remains the same as in the preceding one in each enzyme (Figure 11A). However, the orientation of the composite helix axis in LonBA is closer to its counterpart in LonB and slightly deviates from the orientation of the corresponding element in both bacterial and mitochondrial LonAs (Figure 11A).

Pore loop-1 is located between the Walker A and Walker B motives. As was noted before, there is a large difference in the number of residues that separate Walker A from Walker B motif in LonBAs and LonAs (82 residues between the conserved Lys103 in the Walker A and the conserved Asp186 in the Walker B motif of *Bs*LonBA, as compared to 60 residues in LonAs). The insertions responsible for this difference are localized in *Bs*LonBA in two areas. One area includes two loops that connect strand β2 with the preceding helix α3 (residues 103–116) and with the following helix α4 (residues 388–391 and 204–208 in *Ec*LonA and *Ton*LonB, respectively), which is, as described above, only a helical turn in the modeled structure of *Bs*LonBA (residues 145–148). The other insertions are found within Pore loop-1 (residues 149–173, 393–404 and 209–232 in *Bs*LonBA, *Ec*LonA and *Ton*LonB, respectively) (Figure 9). On the other hand, there is a five-residue deletion in *Bs*LonBA in the loop connecting helix α5 (residues 174–178) and strand β3 (residues 182–187) comprising residues of Walker B motif, as compared to the corresponding fragment of *Ec*LonA (residues 411–418). This fragment of *Bs*LonBA is more similar to the corresponding one in *Ton*LonB (Figure 9).

Pore loop-2 in *Bs*LonBA is very short, similar to its counterpart in *Ton*LonB. It contains only two residues 191–192, unlike the corresponding Pore loop-2 in *Ec*LonA that comprises eight residues (428–434) (Figure 9, and Figure 11A). In *Ec*LonA, this loop is more extended into the central pore, enabling interactions with the substrate.

**Figure 12:**
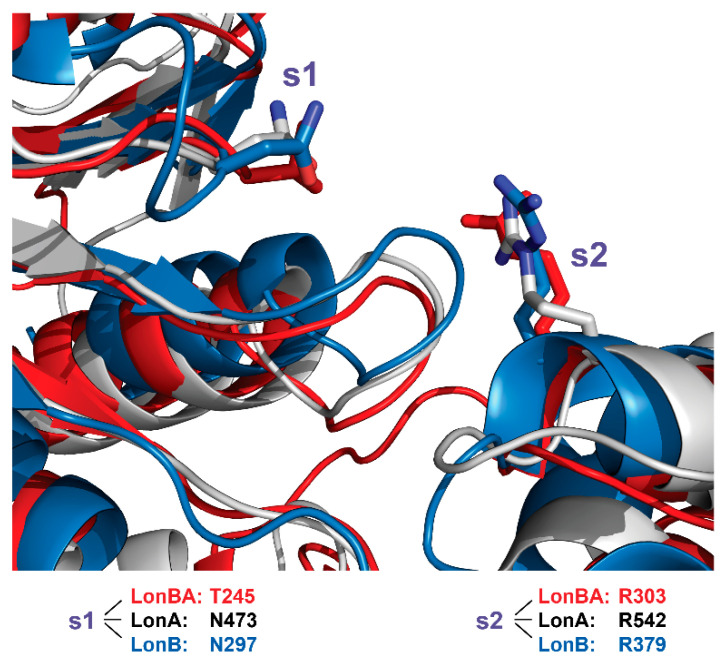
Sensor-1 and sensor-2 residues. Superposition of the ATPase domains of *Bs*LonBA (red), *Ec*LonA (white), and *Ton*LonB (blue) reveals the structurally equivalent placement of the s1 and s2 residues in all three enzymes.

The RKH loop is the longest in *Bs*LonBA (residues 206–238), its length closer to its counterpart in *Ton*LonB (residues 265–290) than to the shorter loop in *Ec*LonA (residues 445–466) (Figure 9, and Figure 11B). The location and surrounding of the sensor-1 and sensor-2 residues, as well as the arginine finger, are highly conserved in all three enzymes (Figure 12).

**Figure 13:**
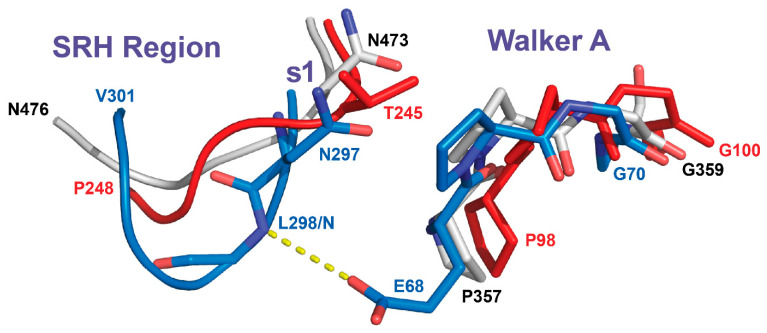
Loop comprising the SRH region (shown as a ribbon) with the s1 residue and the Walker A motif (both shown in sticks and marked). Disposition of the two fragments comprising the SRH region and the Walker A motif in *Bs*LonBA (red), *Ec*LonA (white), and *Ton*LonB (blue) is compared.

On the other hand, the loop comprising the SRH region with sensor-1 residue and the Walker A motif in *Bs*LonBA are more similar to their counterparts in *Ec*LonA. In *Ton*LonB these two fragments bear small differences. The fragment comprising the SRH region with the s1 residue is longer than in both *Bs*LonBA and *Ec*LonA. Additionally, the conserved Pro residue in the Walker A motif (Pro98 in *Bs*LonBA and Pro357 in *Ec*LonA) is substituted by Glu68 in *Ton*LonB. As a result, the longer SRH loop in the latter adopts a different conformation that permits the NH group of Leu298 (the next residue after s1) to interact with the side chain of Glu68 (Figure 13).

### 2.8. A Model of the Full-Length BsLonBA

**Figure 14:**
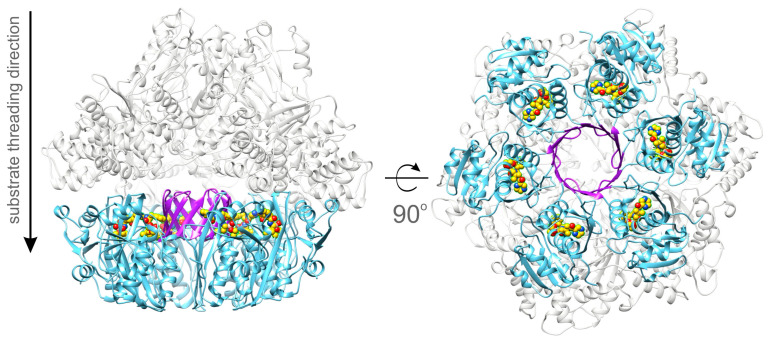
Predicted model of the *Bs*LonBA hexamer. The model represents the experimental hexameric crystallographic assembly of the P domains (light blue) and an approximate, possible organization of the A domains (gray). A model of the A domain was generated in AlphaFold; coordinates of the P domain assembly and six copies of the predicted A domain were superposed onto the structure of *Ton*LonB (3k1j). The central 12-stranded β-barrel created by β4–β5 hairpins of the six protease domains is depicted in violet.

A putative model of the almost full-length *Bs*LonBA was obtained by superposition of the hexamer of the experimentally determined structures of its P domains on the hexamer of the full-length crystal structure of *Ton*LonB (3k1j), followed by individual superposition of each AAA^+^ module (generated with AlphaFold2, with residues 1–56 truncated) on each AAA^+^ module of 3k1j. The resulting model is shown in Figure 14. However, since the crystal structure of *Ton*LonB is quasi-symmetric in the absence of a bound substrate, it should be stressed that the resulting model of *Bs*LonBA should be considered tentative, as structures of substrate-free, full-length members of the LonA subfamily are helical rather than planar. Nevertheless, this modeling exercise indicates that the core part of *Bs*LonBA could resemble the structures of other members of the Lon family, although this still needs experimental verification. At this time, there are no data indicating whether the N-terminal fragment of *Bs*LonBA is indeed folded into a helical conformation and whether it serves as a membrane anchor.

This model of the full-length *Bs*LonBA suggests that a protein substrate threaded through the axial channel (A-pore) of the AAA^+^ module likely interacts with the external side of the unique barrel in the P domain hexamer formed by the β4–β5 hairpins (Figure 4). In addition, threading of the substrate through the barrel seems rather unlikely, because the substrate would miss the proteolytic active site. Moreover, the polar interior of the barrel (Figure 5) would act repulsively against bulky hydrophobic side chains of the processed substrate. In the generated model of the full-length *Bs*LonBA, there is a ~20 Å gap between the bottom part of the A-pore and the inserted barrel. This provides enough space for the threaded substrate to be translocated to one of the proteolytic sites where it could be processed.

## 3. Materials and Methods

### 3.1. Analysis of the Amino acid Sequences of Lon Proteases

The amino acid sequences of the chaperones and proteases analyzed in this study were obtained from the UniProt Knowledgebase (http://www.uniprot.org/, accessed on 20 May 2022) and the MEROPS database (https://www.ebi.ac.uk/merops/, accessed on 20 May 2022).

For secondary structure prediction the following programs were used: Porter 5.0 (http://distilldeep.ucd.ie/porter/, accessed on 20 May 2022), PSSpred (https://zhanggroup.org/PSSpred/, accessed on 20 May 2022), Jped4 (http://www.compbio.dundee.ac.uk/jpred4/index.html, accessed on 20 May 2022) and RaptorX (http://raptorx.uchicago.edu/StructurePropertyPred/predict/, accessed on 20 May 2022).

The search for transmembrane fragments in protein sequences was carried out using programs SMART (http://smart.embl-heidelberg.de/smart/set_mode.cgi?NORMAL=1, accessed on 20 May 2022), TMHMM (https://services.healthtech.dtu.dk/service.php?TMHMM-2.0, accessed on 20 May 2022), HMMTOP (http://www.enzim.hu/hmmtop/html/submit.html, accessed on 20 May 2022) and MPEx (https://blanco.biomol.uci.edu/mpex/, accessed on 20 May 2022).

ExPASy server (www.expasy.org), PSIPRED (http://bioinf.cs.ucl.ac.uk/psipred, accessed on 20 May 2022), and CoCoPRED (http://www.csbio.sjtu.edu.cn/bioinf/CoCoPRED/, accessed on 20 May 2022) were used to identify the coiled-coil regions in proteins and for the subsequent topology analysis. The alignments of the protease sequences were performed using the Clustal Omega program (https://www.ebi.ac.uk/Tools/msa/clustalo/, accessed on 20 May 2022).

### 3.2. Molecular Cloning, Purification, Characterization, and Limited Proteolysis of the Recombinant BsLonBA

The gene encoding *Bs*LonBA was amplified from the genome of *B.s subtilis* strain 168 using a pair of primers: the forward primer (5′-CGTACTACCTGCACCTCATGAGTTGGACAGGGATCGC-3′) and the reverse one (5′- GTCCTACCTGCATCCTCGAGAACGGATTCTTTATTGATTTCGATATG-3′). Both primers contained a BspMI site (underlined). After digestion with BspMI, the product was purified and ligated into the NcoI/XhoI sites of the isopropyl β-D-thiogalactoside (IPTG)-inducible *E. coli* expression vector pET28a(+) (Novagen, USA). The target form of *Bs*LonBA protease contained an octapeptide (LEHHHHHH) at its C terminus (*Bs*LonBA-H6). The construct was verified by DNA sequencing. *E. coli* strain BL21(DE3) (Novagen, USA) was transformed with the recombinant plasmid. Cells containing expression plasmid were grown to OD_600_ ~ 0.5 at 37 °C in 2YT broth containing 50 µg/mL kanamycin. Production of *Bs*LonBA-H6 was induced with 0.1 mM IPTG for 4 h at 20–25 °C. The protein was purified to homogeneity from cleared cell lysate by sequential chromatography on Ni-Sepharose, Heparin-Sepharose, and Sephacryl S-300 columns (GE Healthcare, Sweden). It should be emphasized that due to the low solubility of *Bs*LonBA-H6 protease, all stages of its isolation were carried out in the presence of 8 mM CHAPS detergent. The final product was judged to be >95% pure by SDS-PAGE. The protein concentration was quantified with Bradford reagent (Bio-Rad, Hercules, CA, USA).

A study of the enzymatic properties of *Bs*LonBA-H6 was carried out in comparison with the corresponding properties of the *Ec*LonA-H6 as described in [54]. The ATPase activity of Lon proteases was tested from the time-dependent accumulation of inorganic phosphate, a product of the ATP hydrolysis [55]. The proteolytic activity was followed electrophoretically by observing the hydrolysis of β-casein as a substrate [56]. The efficiency of the peptidase centers of Lons was estimated by hydrolysis of a low-molecular-weight substrate, the thiobenzyl ester of the *N*-substituted tripeptide (Suc-Phe-Leu-Phe-SBzl, PepTBE). The accumulation of the reaction product, benzyl thiolate, was recorded by its interaction with DTDP, leading to the formation of thiopyridone, the level of which was determined spectrophotometrically [57]. Due to the high propensity of *Bs*LonBA-H6 to precipitation, the basic conditions for the determination of all types of enzymatic activity were: 50 mM Tris pH 8.3, 200 mM NaCl buffer containing CHAPS, and the temperature of 30 °C (See the Supplement).

Limited proteolysis of *Bs*LonBA-H6 with chymotrypsin (Millipore-Sigma, Burlington, MA, USA) was performed in the absence or presence of ATP-Mg^2+^, following the previously described procedures [41]. The common solution consisted of 50 mM Tris pH 8.5, 200 mM NaCl and 8 mM CHAPS, with 0.8–1 mg/mL *Bs*LonBA-H6 and 2 µg/mL chymotrypsin. In another set of experiments, these solutions were supplemented with 5 mM ATP and 20 mM MgCl_2_. All measurements were performed at 25 °C for 1.5 h and were monitored using SDS-PAGE. The boundaries of the chymotryptic fragments of *Bs*LonBA-H6 were determined by N-terminal sequencing.

### 3.3. Cloning, Protein Expression, and Purification of the Protease Domain of BsLonBA

The P domain of *Bs*LonBA, residues Gly347-Val552, was amplified from the full-length gene using the polymerase chain reaction (PCR) with the forward primer 5′-GGGGACAAGTTTGTACAAAAAAGCAGGCTCGGAGAACCTGTACTTCCAGGGTGTTGAACCACAGGTTGGCATTG-3′ and reverse primer 5′-GGGGACCACTTTGTACAAGAAAGCTGGGTTATTAAACGGATTCTTTATTGATTTCGATATGAAACGG-3′. The PCR amplicon was recombined into the Gateway^®^ cloning vector pDONR221 (Thermo Fisher Scientific, USA) and the nucleotide sequence was confirmed by DNA sequencing. The open reading frame encoding the *Bs*LonBA P domain, now with a recognition site for tobacco etch virus (TEV) protease (ENLYFQ/G) fused in-frame to its N terminus, was moved by recombination into the destination vector pDEST-527 (Protein Expression Laboratory, Frederick National Laboratory for Cancer Research, Frederick, Maryland, USA) to produce pJT519. This plasmid directs the expression of the *Bs*LonBA P domain (G347-V552) with an N-terminal hexahistidine tag and an intervening TEV protease recognition site. The fusion protein was expressed in the *E. coli* strain BL21(DE3) (Invitrogen Thermo Fisher Scientific, Waltham, MA, USA). Cells containing expression plasmid were grown to mid-log phase (OD_600_~0.5) at 37 °C in LB broth containing 100 µg/mL ampicillin and 0.2% glucose. Overproduction of fusion protein was induced with 1 mM IPTG for 4 h at 30 °C. The cells were pelleted by centrifugation and stored at −80 °C.

All purification steps were performed at 4–8 °C. *E. coli* cell paste expressing the P domain was suspended in ice-cold 50 mM Tris pH 7.5, 200 mM NaCl, 25 mM imidazole buffer (buffer A) containing cOmplete^®^ EDTA-free protease inhibitor cocktail tablets (Millipore-Sigma, Burlington, MA, USA). The cells were lysed with an APV-1000 homogenizer (SPX Corporation, Charlotte, NC, USA) at 9500–10,000 psi, and centrifuged at 30,000× *g* for 30 min. The supernatant was filtered through a 0.45 µm polyethersulfone membrane and applied to a 5 mL HisTrap FF column (Cytiva, Marlborough, MA, USA) equilibrated in buffer A. The column was washed to baseline with buffer A and then eluted with a linear gradient of imidazole to 500 mM. Fractions containing recombinant protein were pooled, concentrated using an Ultracel^®^ 10 kDa ultrafiltration disc (Millipore-Sigma, Burlington, MA, USA), diluted with 50 mM Tris pH 7.5, 200 mM NaCl buffer to reduce the imidazole concentration to ~25 mM, and digested overnight with His_7_-tagged TEV protease [58]. The digest was applied to a 10 mL HisTrap FF column (2 × 5 mL columns in series) equilibrated in buffer A and the *Bs*LonBA P domain emerged in the column effluent. The effluent was incubated overnight at 4 °C with 10 mM dithiothreitol, concentrated as above, and applied to a HiPrep 26/60 Sephacryl S-200 HR column (Cytiva, USA) equilibrated in 25 mM Tris pH 7.5, 200 mM NaCl, 2 mM TCEP buffer. The peak fractions containing recombinant protein were pooled and concentrated to 9–10 mg/mL (estimated from the absorbance at 280 nm using a molar extinction coefficient of 4470 M^−1^ cm^−1^ derived using the ExPASy ProtParam tool [59]). Aliquots were flash-frozen in liquid nitrogen and stored at −80 °C. The final product was judged to be >95% pure by SDS-PAGE. The molecular weight was confirmed by electrospray ionization mass spectroscopy.

### 3.4. Crystallographic Procedures

Samples of the P domain of *Bs*LonBA (residues 347–552) were mixed with the inhibitor, bortezomib (Millipore-Sigma, Burlington, MA, USA), at the molar ratio 1:6 in a buffer consisting of 20 mM Tris pH 7.5, 200 mM NaCl, and 2 mM TCEP. Subsequently, 10 mM MgCl_2_ was added to the sample which was then concentrated to 46.4 mg/mL. Crystals were grown by the hanging drop method, by mixing 1 µL of sample with an equivalent amount of mother liquor consisting of 35% PEG400 at pH 7.8. Hexagonal plate crystals appeared in three days and fully grew to the size of 0.2 × 0.2 × 0.05 mm in 2 weeks. We were unable to grow crystals in the absence of bortezomib.

Diffraction data for the crystals with bound inhibitor were collected at the SER-CAT beamline ID-22 of the Advanced Photon Source, Argonne National Laboratory. The crystal used for data collection was flash cooled in liquid nitrogen without any additional cryoprotectant. Data were indexed and integrated with XDS [60] and scaled with Aimless [61] (Table 1). The structure was solved by molecular replacement using the structure of the P domain of *Ec*LonA [34] (PDB ID 1rre) as a starting model. Subsequently, the structure was rebuilt in Phenix AutoBuild [62] and refined in Coot [63] and Refmac5 [64]. TLS parameters [65,66] were applied at a later stage of structure refinement. The quality of refined structures was controlled by factors R_work_ and R_free_ [67] and by geometric parameters. Procheck [68] and MolProbity [69] were used for the evaluation of the final model. Residues 352–547 could be modeled in the electron density maps for chain A, whereas we were able to trace only residues 348–535 in chain B. The difference, especially in the C-terminal part, is caused by the contacts with neighboring subunits in the crystal lattice. The final refinement statistics are presented in Table 1.

### 3.5. Modeling Procedures

The amino acid sequences of the ATPase (A) domain and the protease (P) domain of *Bs*LonBA were submitted individually to the AlphaFold2 [70] ColabFold server (https://colab.research.google.com/github/sokrypton/ColabFold/blob/main/AphaFold2-.ipynb, accessed on 5 April 2022) and the program was run with default parameters. The highest-ranked model of each domain was used for comparisons with the experimental structure of the P domain and for the construction of a model of the hexamer. A comparison of the AlphaFold2 model of the P domain with the subsequently determined crystal structure resulted in an rmsd of 1.3 Å, indicating high accuracy of the prediction and, by extrapolation, suggesting that the prediction of the structure of the A domain might also be trustworthy.

A putative model of a hexamer of *Bs*LonBA was obtained by superposition of the hexamer of the P domains created by application of crystallographic symmetry to the experimental coordinates on the hexamer of *Ton*LonB, also obtained by applying the crystal symmetry operators to the coordinate set 3k1j [71]. A model of the A domain obtained with AlphaFold2 was superimposed on each A domain of the target hexamer in turn, after truncating residues 1–56 that were modeled as a single helix. The N-terminal part of the predicted structure includes a helix that might be inserted into the membrane and thus anchor this protease.

Figure 2, Figure 3, Figure 4, Figure 5 and Figure 6 were created with the program Chimera [72], whereas Figure 7, Figure 8 and Figure 10, Figure 11, Figure 12, Figure 13 and Figure 14 were drawn with PyMol [73]. Coordinates were aligned with the programs Align [74] and/or PyMol [73].

## 4. Conclusions

### A Proposal to Assign LonBA as a Separate Lon Subfamily

Based on the analysis of the primary through quaternary structure of *Bs*LonBA presented here, we propose that members of this subfamily of firmicute Lon proteases should be transferred from their current assignment in databases such as MEROPS, in which they share their designation with archaeal LonB enzymes. Although LonBAs are presumably membrane-bound, the insertion domain is located at their N terminus, thus differentiating them from LonB enzymes. Additionally, as discussed in detail above, some functionally important elements in the ATPase domain bear more resemblance to LonA, and the others to LonB. The catalytic centers of the protease domains resemble LonAs more than LonBs. An insertion that creates a unique 12-stranded β barrel in the protease domain of LonBA is not found in any other Lons. These features differentiate LonBAs from the already established A and B families of Lons. More complete characterization of the LonBA subfamily will require further studies of the biochemical properties and the biological role of these enzymes, as well as obtaining structural data for the full-length LonBAs.

## Figures and Tables

**Table 1:** Data collection and refinement statistics.

Data Collection	LonBA
Space group	*P*6_3_
Unit cell parameters a, b, c (Å) α, β, γ (˚)	89.9, 89.9, 83.95 90, 120, 90
Temperature (K)	100
Wavelength (Å)	1.000
Resolution (Å)	84–1.9 (1.94–1.90)
R_merge_ (%)	4.4 (82.7)
Completeness (%)	99.9 (99.0)
Mean I/σ(I)	30.1 (1.8)
Total reflections	294,057 (10,969)
Unique reflections	30,425 (1942)
Redundancy	9.7
CC1/2	1.000 (0.797)
B factor from Wilson plot (Å^2^)	39.6
No. of molecules in a.u.	2
**Refinement statistics**	
Resolution (Å)	30–1.9 (1.95–1.90)
Working set: no. of reflections	28,500
R_factor_ (%)	19.4 (31.6)
Test set: no. of reflections	951
R_free_ (%)	23.7 (34.2)
Protein atoms	2831
Solvent atoms	159
Ligand atoms	58
Geometry statistics	
R.m.s.d. bond distance (Å)	0.01
R.m.s.d. bond angle (°)	1.65
Isotropic average B-factor (Å^2^)	47.0
Ramachandran plot (%)	
Favored regions	97
Allowed regions	3
Disallowed regions	0
PDB ID	8dvh

## Data Availability

Coordinates and structure factors have been deposited in the Protein Data Bank with the accession number 8dvh.

## Supplementary Materials

The supporting information can be downloaded at: https://www.mdpi.com/article/10.3390/ijms231911425/s1.

## Abbreviations

AAA^+^, **A**TPases **A**ssociated with diverse cellular **A**ctivities; ATP, adenosine triphosphate; PepTBE, Succinyl-Phe-Leu-Phe-SBzl; SDS–PAGE, sodium dodecyl sulfate–polyacrylamide gel electrophoresis; TCEP–Tris(2-carboxyethyl)phosphine; cryo-EM, cryo-electron microscopy; MA, membrane anchoring; MTS, mitochondial targeting sequence; NTD, N-terminal domain; PDB, Protein Data Bank; PQC, protein quality control; *Ec*LonA, *Escherichia coli* LonA; *Bs*LonA, *Bacillus subtilis* LonA; *Mta*LonA, *Meiothermus taiwanensis* LonA; *h*MtLonA, Human mitochondrial LonA; *Tt*LonA, *Thermus thermophilus* LonA; *Af*LonB, *Archaeoglobus fulgidus* LonB; *Ton*LonB, *Thermococcus onnurineus* LonB; *Mj*LonB, *Methanocaldococcus jannaschii* LonB; *Mta*LonC, *M. taiwanensis* LonC.
