# Supplementary material for: Unique Structural Fold of LonBA Protease from Bacillus subtilis, a Member of a Newly Identified Subfamily of Lon Proteases
Source: Int J Mol Sci. 2022 Sep 28;23(19):11425. doi: 10.3390/ijms231911425 (PMC9569914; doi:10.3390/ijms231911425)
Supplement: Supplementary file 1 [file ijms-23-11425-s001.zip › ijms-1899257-supplementary.pdf]

Supplementary Material

Unique structural fold of LonBA protease from *Bacillus subtilis*, a member of a newly identified subfamily of Lon proteases

Alla Gustchina[1], Mi Li[1,2], Anna G. Andrianova[3], Arsen M. Kudzhaev[3], George Lountos[1,2], Bartosz Sekula[1,4], Scott Cherry[1], Joseph E. Tropea[1], Ivan V. Smirnov[3], Alexander Wlodawer[1]*, Tatyana V. Rotanova*[3]

[1]Center for Structural Biology, National Cancer Institute, Frederick, MD 21702, USA

[2]Basic Science Program, Leidos Biomedical Research, Frederick National Laboratory for Cancer Research, Frederick, MD 21702, USA

[3]Shemyakin-Ovchinnikov Institute of Bioorganic Chemistry, Russian Academy of Sciences, Moscow, 117997, Russia

[4]Institute of Molecular and Industrial Biotechnology, Lodz University of Technology, Lodz, 90-573, Poland

```
                 Chymotrypsin ▼    |←                    NTD3H  subdomain                →|←      Linker-1      →|  α/β subdomain →
                 (M234-K235)  ▼        250        260        270        280        290        300        310        320        330        340        350
EcLonA    ◄ N and NTD5H subdomains ► DAPDENEALKRKIDAAKMPKEAKEKAEAELQKLKMMSPMSAEATVVRGYIDWMVQVPWNARSKVKKDLRQAQEILDTDHYGLERVKDRILEYLAVQSRVNKIKGPIL   352
BsLonA    ◄ N and NTD5H subdomains ► GKTGEVQTLTEKIEEAGMPDHVKETALKELNRYEKIPSSSAESSVIRNYIDWLVALPWTDETDDKLDLKEAGRLLDEEHHGLEKVKERILEYLAVQKLTKSLKGPIL   350
BcLonA    ◄ N and NTD5H subdomains ► GKGGEVEELREKIEQSGMPEETMKAALKELDRYEKLPVSSAESGVIHNYIDWLLALPWTTATEDMIDLTHSEEILNKDHYGLEKVKERVLEYLAVQKLTNSLKGPIL   352
BhLonA    ◄ N and NTD5H subdomains ► GKTGEVASLREQIDEANMPENVEEKALKELERYEKMPASSAESSVLRNYLDWLIQLPWVKETEDILDVNRAERILDEDHYGLEKVKERVIEYLAVQQLTKELKGPIL   351
BmLonA    ◄ N and NTD5H subdomains ► GKGGEVEELREKIEQSGMPEETMKAALKELDRYEKLPVSSAESGVIHNYIDWLLALPWTTATEDMIDLTHSEEILNKDHYGLEKVKERVLEYLAVQKLTNSLKGPIL   352
BpLonA    ◄ N and NTD5H subdomains ► GKTGEVSSLMSKIEESSMPDSVRETALKELNRYEKIPSSSAESSVIRNYIDWLIGLPWGIYTEDRLDLKIASEILDDEHHGLEKVKERVLEYLAVQKLTNSLKGPIL   350
BbLonA    ◄ N and NTD5H subdomains ► GRQGEVDELRAGLEKSDAPERIKAKIEKELERLEKMPATSAEGSVIRTYIDTLFALPWTKTTEDNLDIHHAQQVLDEDHYGLDKPKERVLEYLAVQKLVNSMRGPIL   345
GtLonA    ◄ N and NTD5H subdomains ► GKTGEVEALKEKIEAAGMPEHVKETALKELDRYEKIPATSAESAVIRNYLDWLIALPWSKETKDIHDIKRAETILNEEHYGLDKVKERVLEFLSVQQLTQSLKGPIL   356
PlLonA    ◄ N and NTD5H subdomains ► GRAGEVEELRSQLQELQLPERVHEKVEKEIDRLEKMPASSAEGGVIRNYVDWLLALPWTNKTEDDLDIAKAEQVLDEDHYGLEKPKERVLEYLAVQKLVKKMKGPIL   352
                                                    10         20         30         40         50         60         70              80         90
BsLonBA-1  --------------------------------MSWTGIALFIQLFFGIIIGLYFWNLLKNQRTQKVTIDKESKKEMEQLRKMRAISLSEPLSEKVRPKSFKDIVGQEDGIK------ALKAALCGPNPQHV   93
BsLonBA-2  --------------------------------MSWTGIALFIQLFFGIIIGLYFWNLLKNQRTQKVTIDKESKKEMEQLRKMRAISLSEPLSEKVRPKSFKDIVGQEDGIK------ALKAALCGPNPQHV   93
BcLonBA    -------------------------------MMSWTNIFLLVQLVFGVIVGLYFWHLLRNQRTQKVSIDRESKKELEQLRKMREISLTEPLAEKVRPTSFLDIVGQEDGIK------SLKAALCGPNPQHV   94
BhLonBA    --------------------------------MNWTGILFVVQLFFGVVIGLYFWNLLKTQRSQRVSIDRESRKEMDQLRKLQSISLSEPLSEKVRPESFEDIVGQKEGIR------ALKAALCGPNPQHV   93
BmLonBA    --------------------------------MSWTNIFLLVQLVFGVIVGLYFWHLLRNQRTQKVSIDRESKKELEQLRKMREISLTEPLAEKVRPTSFLDIVGQEDGIR------SLKAALCGPNPQHV   93
BpLonBA    --------------------------------MSWTGIVLFIQLFFGIVIGLYFWNLLRNQRTQKVTIDKESKKEMEALRKMRSIKLSEPLSERVRPKGFQDIVGQEDGIR------ALKAALCGPNPQHV   93
BbLonBA    -------------------------------MDYTTLVIAVIEVVVGIVIGTYFWNLLRAQRNTKTSTEKESRKDLDSIRKMRMVALTEPLSERTRPATLEEIVGQEDGLR------ALRAALCGPNPQHV   94
GtLonBA    --------------------------------MNWTNIVLVIQLFFGVVIGLYFWNLLKGQRVQKVSIDKESRKEMEQLRKLRSISLTEPLAEKVRPKSFDDIVGQEDGIK------ALKAALCGPNPQHV   93
PlLonBA    --------------------------------MNLSIVIMLVQLFFAVIIGIYFWNLLRGQKTSKTAVDRESRKELDKLRKLRSVSLTKPLAEKTRPQTMDDIVGQKDGLR------ALKAALCSANPQHV   93
```

```
                                                        α/β subdomain
           Walker A                                     Pore loop-1                              Walker B  Pore loop-2        RKH loop
                360        370                   380           390   ↓ 400                   410        420        430        440        450
EcLonA     CLVGPPGVGKTSLGQSIAKAT----------GRKYVRMALG-----GVRDEAEIRG--HRRTYIGSMP-----------GKLIQKMAKVGVKNPLFLLDEIDKMSSDMRGDPASALLEVLDPEQNVAFSDH   455
BsLonA     CLAGPPGVGKTSLAKSIAKSL----------GRKFVRISLG-----GVRDESEIRG--HRRTYVGAMP-----------GRIIQGMKKAGKLNPVFLLDEIDKMSSDFRGDPSSAMLEVLDPEQNSSFSDH   453
BcLonA     CLVGPPGVGKTSLARSIATSL----------NRNFVRVSLG-----GVRDESEIRG--HRRTYVGAMP-----------GRIIQGMKKAKTVNPVFLLDEIDKMSNDFRGDPSAALLEVLDPEQNHNFSDH   455
BhLonA     CLAGPPGVGKTSLARSVARAL----------NREFVRISLG-----GVRDEAEIRG--HRRTYVGAMP-----------GRIIQGMKKAGSVNPVFLLDEIDKMANDFRGDPAAALLEVLDPEQNHTFSDH   454
BmLonA     CLVGPPGVGKTSLARSIATSL----------NRNFVRVSLG-----GVRDESEIRG--HRRTYVGAMP-----------GRIIQGMKKAKTVNPVFLLDEIDKMSNDFRGDPSAALLEVLDPEQNHNFSDH   455
BpLonA     CLAGPPGVGKTSLAKSIAKSL----------DRKFIRVSLG-----GVRDESEIRG--HRRTYVGAMP-----------GRIIRGMSKAGTMNPVFLLDEIDKMSSDFRGDPSSAMLEVLDPEQNHNFSDH   453
BbLonA     CLVGPPGVGKTSLARSVARAI----------GREFVRISLG-----GVRDEAEIRG--HRRTYVGALP-----------GRIIQGMKQAGTVNPVFLLDEIDKLASDFRGDPASALLEVLDPNQNDKFSDH   454
GtLonA     CLAGPPGVGKTSLARSIAKAL----------GRRFVRMSLG-----GVRDESEIRG--HRRTYVGAMP-----------GRIIQGMKKAGTINPVFLLDEIDKMSSDFRGDPSAAMLEVLDPEQNHTFSDH   458
PlLonA     CLVGPPGVGKTSLARSIARSL----------ERKFVRISLG-----GVRDEAEIRG--HRRTYVGAMP-----------GRIIQGMKTAGSLNPVFLLDEIDKMASDFRGDPSSALLEVLDPEQNNTFSDH   454
               100        110        120        130        140        150   ↓  160        170          180          190            200        210
BsLonBA-1  IVYGPPGVGKTAAARLVLEEAKKHKQSPFKEQAVFVELDATTARFDERGIADPLIGSVHDPIYQGAGAMGQAGIPQPKQG-AVTHA--HGG---VLFIDEIGE---LH--PIQMNKMLKVLEDRKVFLDSA   213
BsLonBA-2  IVYGPPGVGKTAAARLVLEEAKKHKQSPFKEQAVFVELDATTARFDERGIADPLIGSVHDPIYQGAGAMGQAGIPQPKQG-AVTHA--HGG---VLFIDEIGE---LH--PIQMNKMLKVLEDRKVFLDSA   213
BcLonBA    IIYGPPGVGKTAAARLVLEEAKRNPKSPFRTNATFIELDATTARFDERGIADPLIGSVHDPIYQGAGAMGQAGIPQPKKG-AVTDA--HGG---ILFIDEIGE---LH--PIQMNKMLKVLEDRKVFLESA   214
BhLonBA    IIYGPPGVGKTAAARLVLQEAKKNPNSPFAHDAVFVELDGTTARFDERGIADPLIGSVHDPIYQGAGAMGQAGIPQPKQG-AVTKA--HGG---MLFIDEIGE---LH--SIQINKLLKVLEDRKVFLESA   213
BmLonBA    IIYGPPGVGKTAAARLVLEEAKKNQKSPFRMNATFIELDATTARFDERGIADPLIGSVHDPIYQGAGAMGQAGIPQPKKG-AVTDA--HGG---ILFIDEIGE---LH--PIQMNKMLKVLEDRKVFLESA   213
BpLonBA    IVYGPPGVGKTAAARLVMEEAKRHADSPFKKDAVFVELDATTARFDERGIADPLIGSVHDPIYQGAGAMGQAGIPQPKQG-AVTHA--HGG---VLFIDEIGE---LH--PIQMNKMLKVLEDRKVFLDSA   213
BbLonBA    IIYGPPGVGKTAAARVVLEEAKKNQLSPFSSDAKFIEIDATIARFDERGIADPLIGSVHDPIYQGAGSLGQAGIPQPKPG-AVTKA--HGG---MLFLDEIGE---LH--PIQMNKLLKVLEDRKVMLESA   214
GtLonBA    IIYGPPGVGKTAAARLVLEEAKKNPLSPFKKNAVFVELDATTARFDERGIADPLIGSVHDPIYQGAGAMGQAGIPQPKQG-AVTNA--HGG---VLFIDEIGE---LH--PIQMNKLLKVLEDRKVFFESA   213
PlLonBA    IIYGPPGVGKTAAARVVMEEAKKNPQSPFKYDAKFTEIDATTARFDERGIADPLIGSVHDPIYQGAGAMGVAGIPQPKPG-AVTKA--HGG---ILFIDEIGE---LH--PIQMNKLLKVLEDRKVLLESA   213
```

```
                                          ← α/β subdomain | α subdomain →
              RKH loop                SRH region
                                 s1↓                 RF↓       ▼Chymotrypsin                                 s2
           460           470          480         ▼      500       510       520       530       540       550       560
EcLonA     YLEVDYD--------------LSDVMFVATSN------S-MNI-PAPLLDRMEVIRLSGYTEDEKLNIAKRHLLPKQIERNALKKGELTVDDSAIIGIIRYYTREAGVRGLEREISKLCRKAVKQLLLDKS    564
BsLonA     YIEETFD--------------LSKVLFIATAN------NLATI-PGPLRDRMEIINIAGYTEIEKLEIVKDHLLPKQIKEHGLKKSNLQLRDQAILDIIRYYTREAGVRSLERQLAAICRKAAKAIVAEER    563
BcLonA     YIEEPYD--------------LSKVMFVATANTLATANTLASV-PGPLLDRMEIISIAGYTELEKVHIAREHLLPKQLKEHGLRKGNLQVRDEALLEIIRYYTREAGVRTLERQIAKVCRKAAKIIVTAER    571
BhLonA     YIEEPYD--------------LSKVMFITTAN------NIMTI-PGPLLDRMEIIQIAGYTEVEKQRIALDYLLPKQMKEHGLTKGTFQVKEEAILKVIRYYTREAGVRSLERQMATLCRKGAKMLVSGKK    564
BmLonA     YIEEPYD--------------LSKVMFVATAN------TLASV-PGPLLDRMEIISIAGYTELEKVHIAREHLLPKQLKEHGLRKGNLQVRDEALLEIIRYYTREAGVRTLERQIAKVCRKAAKIIVTAER    565
BpLonA     YIEETFD--------------LSQVLFIATAN------NLATI-PGPLRDRMEIITIAGYTEVEKAEIVKDHLLPKQLKEHGLKKGNLQLREAAIYDTIRYYTREAGVRGLERQLAAICRKAARAIVAEDR    563
BbLonA     YIEETYD--------------LTNVMFITTAN------SLDTI-PRPLLDRMEVISISSYTELEKLNILRDYLLPKQMQDHGLGKDKLKMNDDAMLKLVRLYTREAGVRNLNREAANVCRKAAKLIVGGEK    564
GtLonA     YIEEPYD--------------LSKVMFIATAN------NLATI-PQPLLDRMEIIHIPGYTEVEKLHIAKRHLLPKQLAEHGLKKAALQVRDDAMLDIIRHYTREAGVRELERQLAAICRKAARLIVSGEK    569
PlLonA     FIEIPFD--------------LSQVMFVTTAN------ALHNI-PRPLLDRMEVLYIPGYTEIEKLQIANRYLLPKQKSEHGLEPEQLQVDEGALLKVIREYTRESGVRNLEQQVAALCRKAAKRIVTNES    565
                          ▼Chymotrypsin                                                         ▼Chymotrypsin
             220       230       ▼ 240 ↓                250       ↓ 260       270       280                290   ▼  300 ↓     310        320
BsLonBA-1  YYSEENTQIPNHIHDIFQNGLPADFRLIGATT------RMPNEIPPAIRSRCLEVFFRELEKDELKTVAKTAADKI------EKNIS----EEGLDLLTSY-TRN--GREAVNMIQIAAGMAVTE---NRK    322
BsLonBA-2  YYSEENTQIPNHIHDIFQNGLPADFRLIGATT------RMPNEIPPAIRSRCLEVFFRELEKDELKTVAKTAADKI------EKNIS----EEGLDLLTSY-TRN--GREAVNMIQIAAGMAVTE---NRK    322
BcLonBA    YYSEENTMIPTYIHDIFQKGLPADFRLVGATT------RSPEEIPPAIRSRCLEVFFRELDTEEIQKVAKNAADKV------EMQIG----ENGIEMIGMY-ARN--GREAINLVQISAGMAINE---ERS    330
BhLonBA    YYSEENQQIPRHIHEIFQRGLPADFRLVGATT------RMPEEIPPAIRSRCLEVYFRSLKREEIVTIAKKAVNRL------GFKAE----EGVYEKISAY-ATN--GRETVNIVQIAAGMATTE---GRH    322
BmLonBA    YYSEENSMIPTYIHDIFQKGLPADFRLVGATT------RSPDEIPPAIRSRCLEVFFRELDTEEIQKVAKNAAEKV------KMEIG----KQGVEMIGMY-ARN--GREAINLVQISAGMAINE---GRS    322
BpLonBA    YYSEENTQIPNHIHDIFQNGLPADFRLIGATT------RTPDEIPPAIRSRCLEIFFKDLDQHELKIVAAKAVQKI------QKELS----DEGLNLLTSY-VKN--GREAVNMVQIAAGMAVTE---NRK    322
BbLonBA    YYSEENNQIPSHIHDVFKYGLPADFRLVGATT------RLPEELPAALRSRCLEIFFRPLKAGEIGCIVRTAVPKM------NMNIE----DSAVSVIERY-ATN--GREAINTLQIAAGIALTE---ERQ    323
GtLonBA    YYSKENPQIPSHIHDIFQNGLPADFRLVGATT------RTPNEIPPAIRSRCLEVFFRELDQDEIALIAKKAAEKI------RLNVS----ESGIRLLAAY-ARN--GREAVNMMQIAAGLAITE---NRE    322
PlLonBA    YYNSEDANTPAYIHDIFQNGLPADFRLVGATT------RSPEEVAPALRSRCMEIFFRPLLPEEIGKIAEDAIQKI------GLGAA----PEAVDVVKQY-CSN--GREAVNMIQLAAGLALTE---KRD    322


          ← α subdomain |←    Linker-2    →| P domain →
                               ▼Chymotrypsin
           570       580       ▼      590      600         610       620           630                       640       650       660       670
EcLonA     LKHIEINGDNLHDYLG----VQRFDYGRADNENRVGQVTGLAWTEVGG--DLLTIETACVPGKGK----LTYTGSL--------------GEVMQESIQAALTVVRARAEKLGINPDFYEKRDIHVHVPEG    671
BsLonA     -KRITVTEKNLQDFIG----KRIFRYGQAETEDQVGVVTGLAYTTVGG--DTLSIEVSLSPGKGK----LILTGKL--------------GDVMRESAQAAFSYVRSKTEELGIEPDFHEKYDIHIHVPEG    669
BcLonA     -KRIVVTEKNIVDLLG----KHIFRYGQAEKTDQVGMATGLAYTAAGG--DTLAIEVSVAPGKGK----LILTGKL--------------GDVMKESAQAAFSYIRSRAEELHIDPDFHEKNDIHIHVPEG    677
BhLonA     -KRVVVTQNTIETMLG----KKRFRYGLAEEQDQVGAATGLAYTTAGG--DTLSIEVSVVPGKGK----LILTGKL--------------GDVMKESAQAAFSYIRSRSKELDIDPSFHEKNDIHIHVPEG    670
BmLonA     -KRIVVTEKNIVDLLG----KHIFRYGQAEKTDQVGMATGLAYTAAGG--DTLAIEVSVAPGKGK----LILTGKL--------------GDVMKESAQAAFSYIRSRAEELHIDPDFHEKNDIHIHVPEG    671
BpLonA     -KRITVTEKNLSEFLG----KRLYRYGQAETTDQVGVVTGLAYTTVGG--DTLSIEVSLSPGKGK----LLLTGKL--------------GDVMRESAQAAFSYIRSKADELNIDPNFNEKHDIHIHVPEG    669
BbLonA     -KRVVVTVKTLETLLG----KPRYRYGLAEIKDQVGSVTGLAWTQAGG--DTLNVEVSILPGKGK----LTLTGKL--------------GDVMKESAQAAFSYIRSRAEQWGIDPAFHEKNDIHIHFPEG    670
GtLonA     -KRVVVTQNNIEEFLG----KRKYRYGQAEAEDQIGVATGLAYTAFGG--DTLAIEVSLAKGNGK----LVLTGKL--------------GDVMKESAQAAFSYVRSRAEELGIDPKFHETYDIHIHVPEG    675
PlLonA     -DSIVIGADDIKDYLG----IPKFRYGMAELEDQVGTVTGLAWTEVGG--ETLTIEVTVVPGTGK----LTLTGKL--------------GDVMKESAQAAFSFTRSKAAELGIDPDFHEKLDVHIHIPEG    671
                      ▼Chymotrypsin
                      ▼         340       350       360       370       380       390       400       410       420                 430
BsLonBA-1  DIT------IEDIEWVIHSSQLTPKHEQKIGVEPQVGIVNGLAVYGPNSGSLLEIEVSVTAAQD--KGSINITGIAEEESIGSQSKSIRRKSMAKGSVENVLTVLRTMG-------MKPSDYDIHINFP-G    437
BsLonBA-2  DIT------IEDIEWVIHSSQLTPKHEQKIGVEPQVGIVNGLAVYGPNSGSLLEIEVSVTAAQD--KGSINITGIAEEESIGSQSKSIRRKSMAKGSVENVLTVLRTMG-------MKPSDYDIHINFP-G    437
BsLonBA-3D                              GVEPQVGIVNGLAVYGPNSGSLLEIEVSVTAAQD--KGSINITGIAEEESIGSQSKSIRRKSMAKGSVENVLTVLRTMG-------MKPSDYDIHINFP-G    437
BcLonBA    FIK------DEDIEWVVHSSQLTPKYEKHIYPIPRIGLVNGLAVYGPNTGALLEIEVTAIKAKD--IGSVNVTGIVEEESIGSQTKSIRRKSMAKGSVDNVLRVLRSLD-------VLPEGYDIHINFP-G    438
BhLonBA    EVT------VADVEWVIHSSQLSPRPERQIPSEPKVGLVNGLAVFGPNTGALLEVEVDVIPSVA--EGSVTITGIAEEEQTGNQARSIRRKSMAKGSVENVLTVLKKMD-------LPIDQYDIHINFP-G    437
BmLonBA    FIK------DEDIEWVVHSSQLTPKYEKRIYPIPKIGLVNGLAVYGPNTGALLEIEVMAIPAAE--KGTINVTGIVEEESIGSQTKSIRRKSMAKGSVDNVLTVLRSLD-------LLPEGYDIHINFP-G    437
BpLonBA    DIT------IEDVEWVIHSSQLTPRYEQKVPEKPKVGVVNGLAVYGPNSGALLEIEVNICKALE--KGSINITGIAEEESIGNQSKSIRRKSMAKGSVENVLTVLRNLG-------IRTNEYDIHINFP-G    437
BbLonBA    DIQ------AADVEWVMHSSQKSPRPEKQVHDTPQVGLVNGLAVYGPNMGSVMELEVTASPAAVPGQGRMAMTGMAEEEEMGSRSRTIRRKSMAKGSIENVLTVLSRMG-------VRPYDYDLHINFP-G    440
GtLonBA    KIL------DKDIEWVIHSSQMAPRYEKKIGSAPAVGVVNGLAVYGPNTGALLEIEVTALPAKG--KGSINVTGIVEEESIGSPEKSVRRKSMARGSAENVITVLRAMG-------VPADRYDIHVNFP-G    434
PlLonBA    SIR------ASDVEWVASSSQLQPRPDRKVPEKPQIGVVNGLAVYGPNMGALLEIEVSAVPVEK-GQGTYNITGVVDEEELGGGSRKLRRKSMAKGSIENVLTVLEAMG-------FSPKDYNLHINFP-G    435
```

```
                                                                                                           ← P domain |
                   *680      690       700       710        *       730       740       750       760       770       780
EcLonA      ATPKDGPSAGIAMCTALVSCLTGNPVRADVAMTGEITLRGQVLPIGGLKEKLLAAHRGGIKTVLIPFENKRDLEEIPDNVIADLDIHPVKRIEEVLTLALQNEPSGMQVVTAK------------------   784
BsLonA      AVPKDGPSAGITMATALVSALTGRAVSREVGMTGEITLRGRVLPIGGLKEKALGAHRAGLTTIIAPKDNEKDIEDIPESVREGLTFILASHLDEVLEHALVGEKK-------------------------   774
BcLonA      AVPKDGPSAGITMATALISALTGIPVSKEVGMTGEITLRGRVLPIGGLKEKTLSAHRAGLTKIILPAENEKDLDDIPESVKENLTFVLASHLDEVLEHALVGVKQ-------------------------   782
BhLonA      ATPKDGPSAGITMATALISALTGRVVKKEVGMTGEITLRGRVLPIGGLKEKSMSAHRAGLTTIIIPKENEKDLEDIPKSVREDLNYILVDHLDQVLKHALGEKK--------------------------   774
BmLonA      AVPKDGPSAGITMATALISALTGIPVSKEVGMTGEITLRGRVLPIGGLKEKTLSAHRAGLTKIILPAENEKDLDDIPESVKENLTFVLASHLDEVLEHALVGVKQ-------------------------   776
BpLonA      AVPKDGPSAGITIATALVSALTGRPVSKEVGMTGEITLRGRVLPIGGLKEKALGAHRAGLKTIILPKDNEKDIDDIPESVREGLTFIPVSHLDEVLEKALVGEGR-------------------------   774
BbLonA      AIPKDGPSAGITMATALVSALTKIPVKKEVGMTGEITLRGRVLPIGGLKEKCMSAHRAGLTTIILPKDNEKDIEDIPESVRGELTFYPVDHLDEVLRHALTKQPVGDKA---------------------   779
GtLonA      AVPKDGPSAGITIATALISALTGKPVSRFVGMTGEITLRGRVLPIGGLKEKTLSAHRAGLKTVILPKDNEKDLADIPDTVKRDLRFVLVSHLDEVLPHALVGWER-------------------------   780
PlLonA      AIPKDGPSAGITIATALISSLTKRHVARDVAMTGEITLRGRVLPIGGLKEKSLAAHRAGYKKILLPKDNERDLRDIPDSVKNDVEFVPVSHMDQVLEHALVEQAGVH-----------------------   778
              440  *    450       460       470       480     * 490       500       510            520       530       540             550
BsLonBA-1   GIPIDGPSAGIAMAAGIFSAIHKIPIDNTVAMTGEISLNGLVKPIGGVIPKIKAAKQSGAKKVIIPYENQQAI---LKQIDG-IEIIAVKTFQEVLDEILVNPPTEQKPFHI-E-----INKE------SV   552
BsLonBA-2   GIPIDGPSAGIAMAAGIFSAIHKIPIDNTVAMTGEISLNGLVKPIGGVIPKIKAAKQSGAKKVIIPYENQQAI---LKQIDG-IEIIAVKTFQEVLDEILVNPPTEQKPFHI-E-----INKE------SV   552
BsLonBA-3D  GIPIDGPSAGIAMAAGIFSAIHKIPIDNTVAMTGEISLNGLVKPIGGVIPKIKAAKQSGAKKVIIPYENQQAI---LKQIDG-IEIIAVKTFQEVLDEILVNPPTEQKPFHI-E-----INKE------SV   552
BcLonBA     GIPIDGPSAGIAMATGVYSAVHHTYVNNEVAMTGEISIHGEVKPIGGVYAKIKAAKKAGAKKVIIPAENMQPF---LYTIKG-IEIIPVRKLKEVFELTFMQENMHRELDIHTTI--DETDAQ------SM   557
BhLonBA     GAPVDGPSAGVAIATGIYSAIKEEKVDNTVAMTGEISIHGTVKPVGGIVAKVEAAKASGAKTVLIPKENEQAI---LQEIEG-ITIIPMKHLDDVLSYALIREETEEETIPANQSFVSAP--A------PV   556
BmLonBA     GIPIDGPSAGIAMATGVYSALHQVYVNNEIAMTGEISIHGEVKPVGGVYAKVKAAKKAGAKKVIIPAENMQPF---LYTIKG-IEIIPVRKLKEVFALTFVQEKLHREIDVHREL--DEGDVQ------SM   556
BpLonBA     GAPIDGPSAGIAMATAIFSAIHQIPIDHLTAMTGEIGLQGQVKPIGGVIPKIKAAKQAGATTVIIPYDNQQSI---LKEIEG-VTIVPVKHFQEVLDQCLVNPPDEKKDGLEEE-----MKRK------SV   553
BbLonBA     GIPVDGPSAGITIATAIYSAIVNQPVDNLLAMTGEVSIHGKVKPVGGVVAKVEAAKQAGATRVLIPQENWQSI---FADMNG-IEIIPVSTVSEVIELAVPQATMQEEEATGFTLQIPEEDLASSPL--SL   565
GtLonBA     GVPVDGPSAGVAIAVGIYSAIYQLPVDHTVAMTGEISIHGYVKPVGGVFAKIKAAKQAGAKKVIIPIENMQSL---LREVSG-IQIIAVRRLEEALIHVFGEETLRRGTAFLPAAAADRSGKK------LV   558
PlLonBA     GTPIDGPSAGIAMATAIVSAIKGIEVDNKIAMTGEMSIHGKVKPIGGVIAKVEAAFQAGATSVIIPKDNWQSL---FQDLSGGLRVIPVETVDEVFRQVFGGSLAADIVELPNTDIYPASSASLL 14 GV   576
```

Figure S1. Alignment of the primary and available or predicted secondary structures of *Ec*LonA and bacillary LonA and LonBA proteases. LonAs and LonBAs are from: *Escherichia coli* (*Ec*LonA, MER0000485, UniProt P0A9M0); *Bacillus subtilis* (*Bs*LonA, MER0000487, UniProt P37945 and *Bs*LonBA, MER0002228, UniProt G4ESU3); *B. cereus* (*Bc*LonA, MER0029007, UniProt J7YLY7 and *Bc*LonBA, MER0029009, UniProt C2NNG6); *B. halodurans* (*Bh*LonA, MER0013686, UniProt Q9K8F6 and *Bh*LonBA, MER0013716, UniProt Q9K8F5); *B. mycoides* (*Bm*LonA, MER0231248, UniProt A0A084IV40 and *Bm*LonBA, MER0399002, UniProt C3AR76); *B. pumilus* (*Bp*LonA, MER0109288, UniProt A8FFV7 and *Bp*LonBA, MER0164590, UniProt A0A8G1JA29); *Brevibacillus brevis* (*Bb*LonA, MER0000486, UniProt C0ZAG5 and *Bb*LonBA, MER0166780, UniProt C0ZAG4); *Geobacillus thermodenitrificans* (*Gt*LonA, MER0090423, UniProt A4IRH0 and *Gt*LonBA, MER0090424, UniProt A4IRH1); *Paenibacillus lactis* (*Pl*LonA, MER0299899, UniProt G4HHX1 and *Pl*LonBA, MER0361527, UniProt G4HHX2).

Domain organization is shown for LonAs. Primary structures were compared (by fragments) using the program https://www.ebi.ac.uk/Tools/msa/clustalo/, fragment boundaries were determined according to the secondary structures. Secondary structures of *Bs*LonBA-1 and *Bs*LonBA-2 were predicted using the programs http://distilldeep.ucd.ie/porter/ and https://colab.research.google.com/github/sokrypton/ColabFold/blob/main/AlphaFold2.ipynb, correspondingly. Secondary structure of *Bs*LonBA P domain (*Bs*LonBA-3D) was determined in this work. Red color indicates amino acids that form α-helices, purple – 3/10 helices, blue – β-strands; amino acids marked in black are not included in the secondary structure elements. Enzyme consensus elements are highlighted in yellow: Walker motifs A and B, Pore loop-1 with the conserved Tyr residue, Pore loop-2, RKH loop, SRH region, the residues sensor-1 (s1), sensor-2 (s2), and Arg finger (RF), catalytic residues Ser and Lys and their environment. Amino acid numbering is shown for the sequences of *Ec*LonA and *Bs*LonBA. ▼ – sites of limited proteolysis by chymotrypsin of *Ec*LonA and *Bs*LonBA.

```
                                                                   | α/β subdomain →                 Walker A
                 10        20        30        40        50        60        70        80        90       100
BsLonBA     1    MSWTGIALFIQLFFGIIIGLYFWNLLKNQRTQKVTIDKESKKEMEQLRKMRAISLSEPLSEKVRPKSFKDIVGQEDGIK------ALKAALCGPNPQHVIVYGPPGVGKTAAA    107
                                            10        20        30        40        50        60                70
TonLonB     1                            MGDNEKINREALAPREYGESLELGIEFTTTEEIEVP-EKLIDQVIGQEHAVEVIKTAANQK--------RHVLLIGEPGTGKSMLG    77
AfLonB      1                                       MNEEVREILGGLDFESTKDVAVP-ERLIDQVIGQDHAVEAIKKAAVQK--------RHVMLIGSPGTGKSMLA    64
MjLonB      1                                               MFSIKFKTTEELPEPSPRLIDQVIGQEEAVKIVLSAVKNK--------RNVILLGDPGVGKSMIV    57
                                                    10        20        30        40                  50        60
MtaLonC     1                                  MRLSYEALEWRTPIENSTEPVSLPPP---PPFFGQERAREALELAIRGG--------FHAYLVGPPSLGKHEAL    63
```

```
                                                                                      Pore loop-1            Walker B  Pore loop-2
                 110                                120       130       140       150       160       170       180       ↓
BsLonBA     108  RLVLEEAKK------------------------HKQSPFKEQAVFVELDATTARFDERGIADPLIGSVHDPIYQGAG-AMGQAGIPQPKQGAVTHAHGGVLFIDEIGELH-P    193
                 80                                 190       200                 210       220       230       240       250
TonLonB     78   QAMAELLP|←  MA insert (86-180)  →|VPKLLVDNCGRTKAPFIDAT-----GAHAGALLGDVRHDPFQSGG-LGTPAHERVEP-GMIHRAHKGVLFIDEIATLS-L    252
AfLonB      65   KAMAELL-|←  MA insert (72-166)  →|VPKLLVDNSDKVTAPFEDAT-----GAHAGALFGDVRHDPFQSGG-LETPAHERVEA-GAIHRAHKGVLYIDEINTLT-I    238
MjLonB      58   KAVGEILS|←  MA insert (66-176)  →|MDLKPVLLYECKKRPLVRAS-----AYNVTRLLGDIKHCPLGGRPPLGTPPHKRIIL-GAIHEAHRGILYVDEIKTMP-L    249
                     70                             260       270                 280       290       300       310       320
MtaLonC     64   LAYLSTQS|← HHE insert (72-250)  →|RPNLLTSSSSGTPPPIVYEP-----YATAPRLFG--RLDYLVDRG-VWSTNVSLIRP-GAVHRAQGGYLILDALSLKREG    321
```

```
                                                                                      ← α/β subdomain | α subdomain →
                                  RKH loop                          SRH region
                      200       210       220       230       240  s1   250       RF    260       270       280
BsLonBA     194  IQMNKMLKVLEDRKVFLDSAYYSEENTQIPNHIHDIFQNGL-PADFRLIGATTRMPNE--IPPAIRSRC----LEVFFRELEKDELKTVAKTAADKI------EKNIS----E    289
                   260       270       280                 290       300       310       320       330       340       350
TonLonB     253  KMQQSLLTAMQEKKFPITGQSEMSSGAMVRTEPV--------PCDFVLVAAGNLDTVD-KMHPALRSRIRGYGYEVYMRTTMPDTIENRRKLVQFVAQEVKRDGKIPHFTKEA    356
AfLonB      239  ESQQKLLTALQDKKFPITGQSERSSGAMVRTEPV--------PCDFILVAAGNLDALM-GMHPALRSRIEGYGYEVYMNDTMPDTPENRQKLVRFVAQEVVKDGKIPHFDKYA    342
MjLonB      250  EVQDYILTALQDKQLPISGRNPNSSGATVETNPI--------PCDFILIMSGNMDDVYNLRAPLL-DRI---DYKIVLKNKMDNTLENRDKLLQFIVQEIKNNN-LNPMTYDG    349
                    330       340       350       360       370            380       390       400                  410
MtaLonC     322  TWEAFKRALRNGQVEPVTEPQAPAGLEVEPFPIQMQVTLVGTPEAFEGLEE-DPAFSE-----LFRIRAEFSPTLPASPENCTALGG-------WLLAQG------FQ-LTQG    414
```

```
                                                      ← α subdomain |           ← Linker →            | P domain →
                 290       300        s2   310       320       330       340       350                 360       370
BsLonBA     290  EGLDLLTSY-TRN--------GREAVNMIQIAAGMAVTE--NRKDIT----IEDIEWVIHSSQL-TPKHEQKIGVEPQV-------------GIVNGLAVYGPNSGSLLEIEV    373
                     360       370       380       390       400          410       420       430       440       450       460
TonLonB     357  VEEIVREAQKRAGRKGHLTLRLRDLGGIVRAAGDIAVKK--GKKYVE----REDVIEAVKMAKP-LEKQLADWYIERKKEYQVIKTEGSEIGRVNGLAVIGEQ--SGIVLPIE    460
AfLonB      343  VAEIIKEARRRAGRRNHLTLRLRELGGLVRTAGDIAKSE--GSDIVR----LEHVLKAKKIAKT-IEEQLADKYIERRKDYKLFITEGYEVGRVNGLAVIGES--AGIVLPII    446
MjLonB      350  CCEVVRIAQYLAGSKDKLTLRLRLLANIIKMANDVAMGKDVEELLGN 22 AEHVRKVFDTGIYSMEKQVALNYIKNFKRYKHIVPNDEPKVGVIYGLAVLGA--GGIGDVTK    478
                      420       430       440       450       460                 470       480       490       500       510
MtaLonC     415  GLTRLYDEARRMAEQRDRMDARLVEIRALAEEAAVLGGGLLTAESVE----QAIAAREHRSFLS--EEEFLRAV---QEGV-IRLRTTGRAVGEVNSLVVVEAA-PYWGRPAR    516
```

```
                     380          390       400       410       420                   430        440   *   450       460       470
BsLonBA    374  SVTAAQD---KGSINITGIAEEESIGSQSKSIRRKSMAKGSVENVLTVLRTMG------------MKPSDYDIHINFP-GGIPIDGPSAGIAMAAGIFSAIHKIPIDNTVAMTG    471
                        470       480                      490       500                 510       520       530       540
TonLonB    461  AVVAPAASKEEGKIIVTGKLG---------------EIAKEAVQNVSAIIKRYKGE----------DISRYDIHVQFLQTYEGVEGDSASISVATAVISALEGIPIRQDVAMTG    549
AfLonB     447  AEVTPSMSKSEGRVIATGRLQ---------------EIAREAVMNVSAIIKKYTGR----------DISNMDVHIQFVGTYEGVEGDSASISIATAVISAIEGIPVDQSVAMTG    535
MjLonB     479  IIVQILESKNPGTHLLN-ISG---------------DIAKHSITLASALSKKLVAEKKLPLPKKDIDLNNKEIYIQFSQSYSKIDGDSATAAVCLAIISALLDIPLKQDFAITG    576
                  520        530                   540       550                560       570          *      590       600
MtaLonC    517  LTARAAPGRDHL-ISIDREAGLGG------------QIFHKAVLTLAGYLRSRYIE---------HGSLPVTISLAFEQNYVSIEGDSAGLAELVAALSAIGNLPLRQDLAVTG    608


                                                                                                                 ← P domain |
                       480      * 490             500            510          520       530       540       550
BsLonBA    472  EISLNGLVKPIGGVIPKIK-------AAKQSGAKKVIIPYENQQ-----AI---LKQIDGIEIIAVKTFQEVLDEILVNPPTEQKPFHIEINKESV                      552
                550      560              570       580            590       600       610       620       630
TonLonB    550  SLSVRGEVLPIGGATPKIE-------AAIEAGIKMVIIPKSNEK-----DVFLSKDKAEKIQIFPVETIDEVLEIALEESEKKRELLRRIRETLPLSL                    635
AfLonB     536  SLSVKGEVLPVGGVTQKIE-------AAIQAGLKKVIIPKDNID-----DVLLDAEHEGKIEVIPVSRINEVLEHVLEDGKKKNRLMSKFKELELAAV                    621
MjLonB     577  SLDLSGNVLAIGGVNEKIE-------AAKRYGFKRVIIPEANMI-----DVIETEG----IEIIPVKTLDEIVPLVFDLDNRGGAERFN                             649
                610       620   *   630       640       650       660       670       680       690       700       710
MtaLonC    609  AVDQTGKVLAVGAINAKVEGFFRVCKALGLSGTQGVILPEANLANLTLRAEVLEAVRAGQFHIYAVETAEQALEILAGARMEGFRGLQEKIRAGLEAFARLEEGHDKEDRE       719
```

Figure S2. Primary and secondary structure alignment of *Bs*LonBA, LonBs and *Mta*LonC proteases based on experimental structures

Enzyme sources: *Bacillus subtilis* – *Bs*LonBA (MER0002228, UNIPROT P42425); *Thermococcus onnurineus* – *Ton*LonB (MER0145010, UNIPROT B6YU74, PDB: 3K1J); *Archaeoglobus fulgidus* – *Af*LonB (MER0003868, UNIPROT O29883, PDB: 1Z0W/B,C,E,G,T,V); *Methanocaldococcus jannaschii* – *Mj*LonB (MER0003359, UNIPROT Q58812, PDB: 1XHK); *Meiothermus taiwanensis* – *Mta*LonC (MER0423045, UNIPROT C9DRU9, PDB: 4FW9/D,G,H,V).
Predicted fragments of structures are shown in the standard font; experimentally determined structures and/or their fragments are highlighted in bold; sequence fragment of *Mta*LonC not seen in the crystal structure is italicized. Amino acid numbering is shown for the sequences of *Bs*LonBA, *Ton*LonB, and *Mta*LonC.
See all designations in the caption to Fig. S1. Substitutions of amino acid residues in Walker motifs are highlighted in gray.

(A)

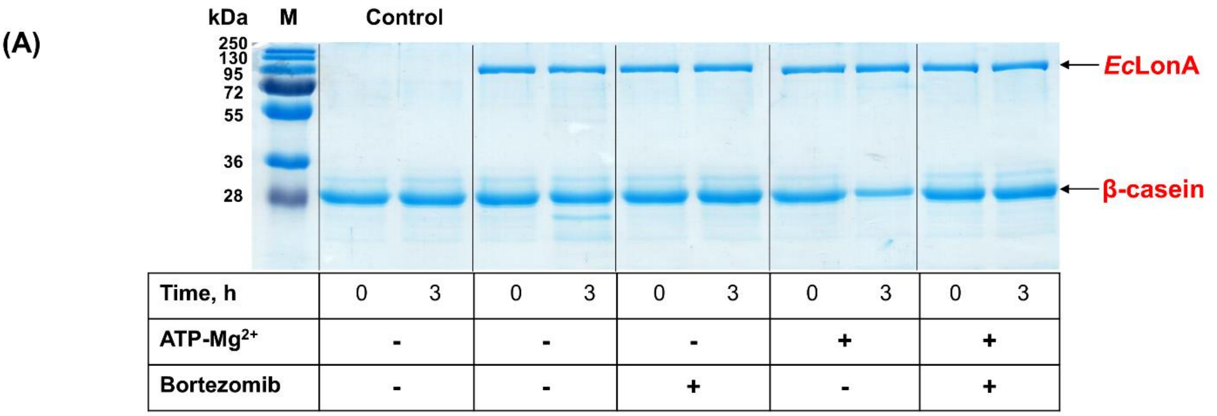

Time, h	0	3	0	3	0	3	0	3	0	3
ATP-Mg^{2+}	-		-		-		+		+	
Bortezomib	-		-		+		-		+	

(B)

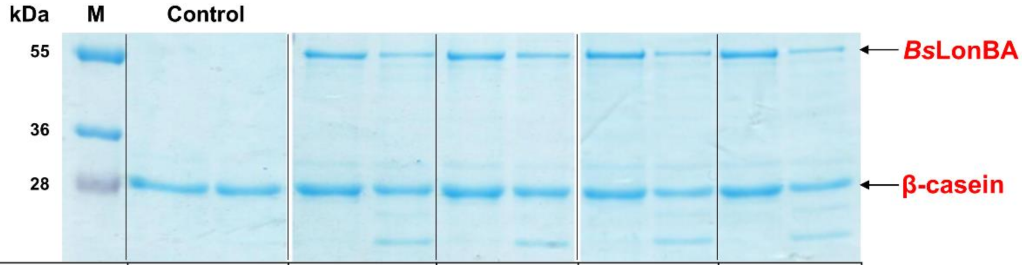

Time, h	0	24	0	24	0	24	0	24	0	24
ATP-Mg^{2+}	-		-		-		+		+	
Bortezomib	-		-		+		-		+	

Figure S3. Hydrolysis of β-casein by *Ec*LonA-H6 (A) and *Bs*LonBA-H6 (B). *Experimental conditions*: 15% PAGE, 50 mM Tris pH 8.3, 200 mM NaCl, 8 mM CHAPS buffer, 1 mM ATP, 10 mM $MgCl_2$, 7.0 (A) or 5.0 (B) µM β-casein, 1.5 µM *Ec*LonA-H6, 3.0 µM *Bs*LonBA-H6, 35 (A) or 70 (B) µM bortezomib; 30 °C. Preincubation of the enzymes with bortezomib was carried out during 1 hour at 25 °C. M, molecular weight markers.

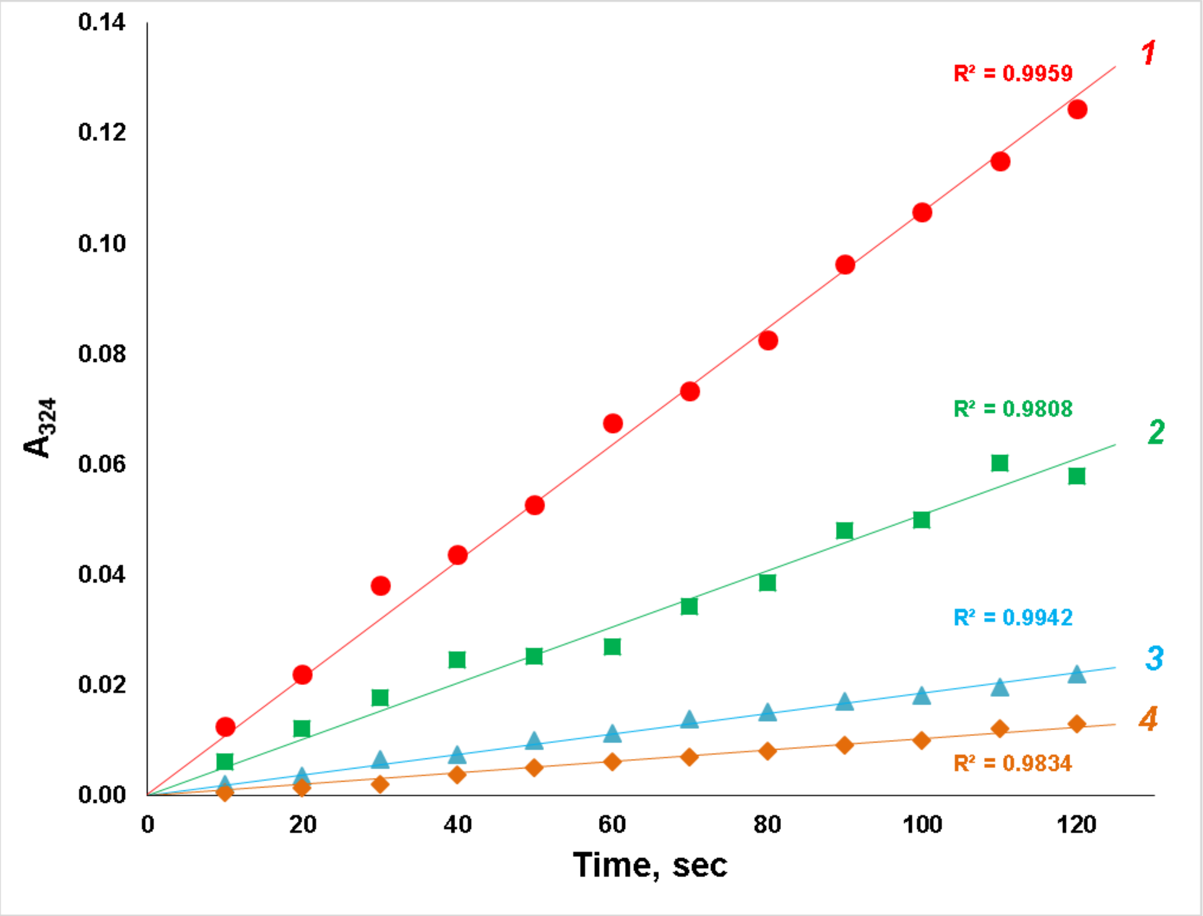

Figure S4. Hydrolysis of PepTBE by *Ec*LonA-H6 (*1*, *2*) and *Bs*LonBA-H6 (*3*, *4*). *Experimental conditions*: 50 mM Tris pH 8.3, 200 mM NaCl, 10% DMSO, 8 mM CHAPS buffer, 0.1 mM PepTBE, 0.2 mM DTDP, 0.5 µM enzymes, 0.05 (*2*) or 1.0 (*4*) µM bortezomib; 30°C. The reactions were initiated by the addition of the substrate.
